# A Concise Asymmetric Synthesis of the Aggregation Pheromone of *Cryptolestes ferrugineus*, Ferrulactone II, and Its Enantiomer

**DOI:** 10.3390/molecules31030404

**Published:** 2026-01-24

**Authors:** Hong Tang, Biyu An, Yiwen Huang, Dan Liu, Qinghua Bian, Jiangchun Zhong

**Affiliations:** Department of Applied Chemistry, China Agricultural University, Beijing 100193, China; thqust@163.com (H.T.); aby19990129@163.com (B.A.); yiwenhuang@cau.edu.cn (Y.H.); dliu@cau.edu.cn (D.L.); bianqinghua@cau.edu.cn (Q.B.)

**Keywords:** *Cryptolestes ferrugineus*, Ferrulactone II, aggregation pheromone, asymmetric synthesis, CBS reduction

## Abstract

A concise and efficient synthesis of the aggregation pheromone of *Cryptolestes ferrugineus*, Ferrulactone II, and its enantiomer has been developed. The synthetic route features CBS reduction, the zipper reaction, a copper(I)-catalyzed coupling, stereoselective partial hydrogenation, Yamaguchi esterification, and the Mitsunobu inversion reaction. The structures and absolute configurations of both enantiomers of the target pheromone were confirmed by NMR, HRMS, specific rotation, and ECD spectroscopy. This study provides a reliable supply of material for further biological evaluation.

## 1. Introduction

The rusty grain beetle, *Cryptolestes ferrugineus* (Stephens), is a serious storage pest found in more than 110 countries on different continents [1,2]. This pest could rapidly multiply and damage wheat, beans, maize, and other cereals, causing great threats to stored grains and significant economic losses [3,4]. Fumigation with phosphine (PH_3_) is the primary strategy to control the rusty grain beetle [5,6]. However, its prolonged, extensive, and inefficient use has led to high levels and widespread phosphine resistance in *C. ferrugineus* [7,8], driving the need for alternative, more efficient, and eco-friendly control [9,10].

**Scheme 1:**
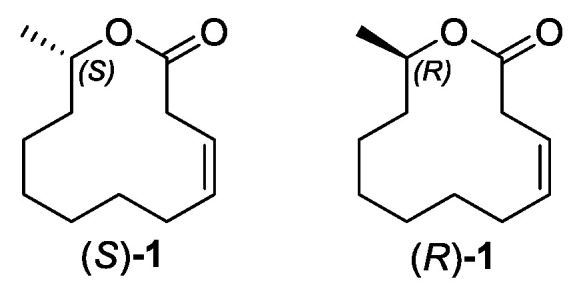
Ferrulactone II and its enantiomer.

Pheromones offer innovative and sustainable approaches to control the stored product pest, with the advantages of being effective, benign to the environment, and rarely inducing pest resistance [11,12]. In 1983, Wong and co-workers identified that the aggregation pheromone of *C. ferrugineus* was (*E*,*E*)-4,8-dimethyl-4,8-decadien-10-olide (Ferrulactone I) and (3*Z*,11*S*)-3-dodecen-11-olide (Ferrulactone II) (Scheme 1), and found that the pheromone could attract male and female rusty grain beetles [13]. Later, Oehlschlager and co-workers used a mixture of the synthetic Ferrulactone I and racemic Ferrulactone II to trap the rusty grain beetle in farm bins [14].

**Scheme 2:**
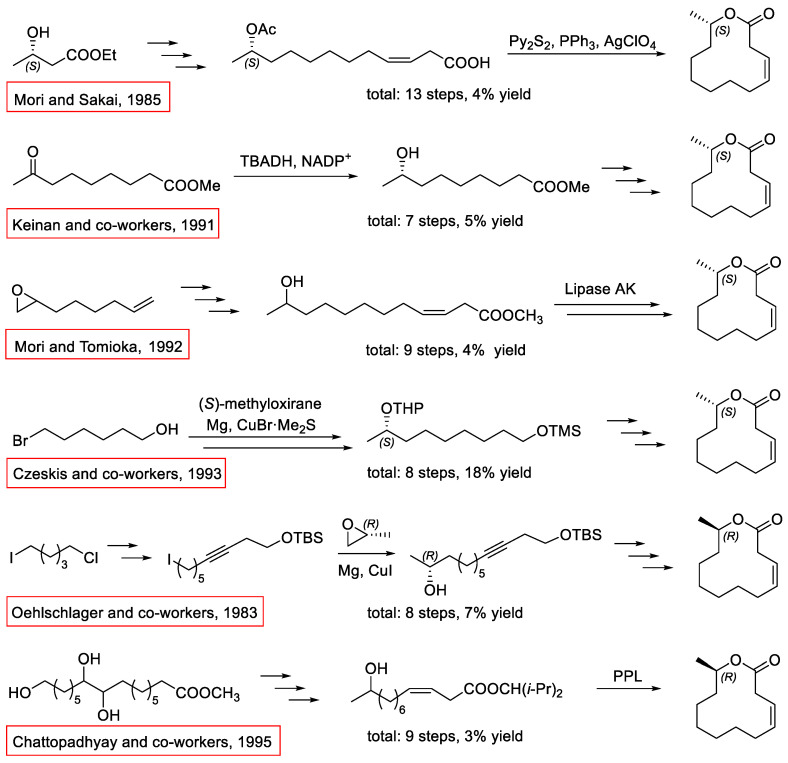
Previous syntheses of Ferrulactone II and its enantiomer [15,16,17,18,19,20].

Due to its significant bioactivity and the application of mass trapping [14], its synthesis has previously been studied by several groups. The previous syntheses of Ferrulactone II and its enantiomer are mainly based on chiral sources of ethyl (*S*)-3-hydroxybutanoate [15] and (*S*)- and (*R*)-methyloxirane [16,17], TBADH catalyzed reduction [18], and Lipase AK or PPL-catalyzed acetylation [19,20]. However, these synthetic strategies have disadvantages such as many steps or low total yields (Scheme 2). Hence, a more concise and efficient asymmetric synthesis of Ferrulactone II and its enantiomer is needed in order to facilitate its implementation as a biorational tool in the management of the rusty grain beetle.

Herein, we report an enantioselective synthesis of Ferrulactone II (*S*)-**1** and its enantiomer (*R*)-**1** (Scheme 1). The key steps of this route include CBS reduction, Yamaguchi esterification, and Mitsunobu-macrolactonization with inversion of the chiral hydroxyl group. Overall, the approach features a concise seven-step sequence with a high overall yield, avoids the use of protecting groups, and enables access to both *S*- and *R*-enantiomers using a single chiral catalyst.

## 2. Results and Discussion

### 2.1. Retrosynthetic Analysis

**Scheme 3:**
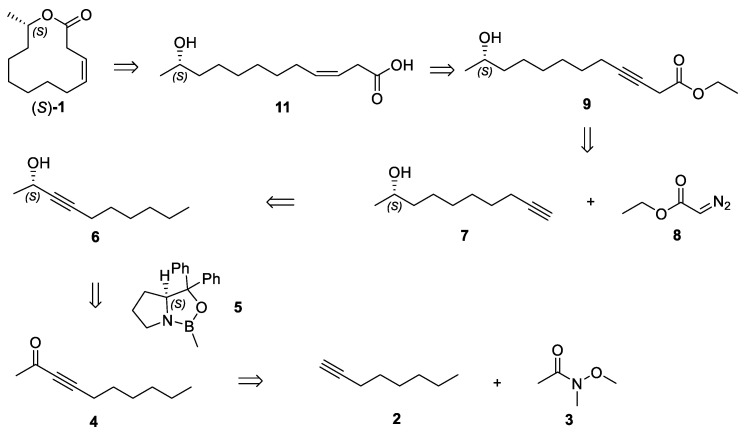
Retrosynthetic analysis of Ferrulactone II (*S*)-**1**.

The retrosynthetic analysis of Ferrulactone II (*S*)-**1** is illustrated in Scheme 3. The target macrolactone (*S*)-**1** could be prepared through Yamaguchi esterification of (*S*,*Z*)-11-hydroxydodec-3-enoic acid (**11**). The *cis* double bond in hydroxyl acid **11** could be generated via partial hydrogenation of (*S*)-11-hydroxydodec-3-ynoate (**9**) catalyzed with Brown’s P2-Ni system. Alkynyl ester **9** could be synthesized by a copper(I)-catalyzed coupling of terminal alkyne **7** with ethyl diazoacetate (**8**). The chiral hydroxy of propargylic alcohol **6** was envisioned to be introduced by an enantioselective CBS reduction of dec-3-yn-2-one (**4**), which could be achieved from Weinreb’s amide **3** and oct-1-yne (**2**).

### 2.2. Synthesis of (S,Z)-11-Hydroxydodec-3-enoic Acid (***11***)

**Scheme 4:**
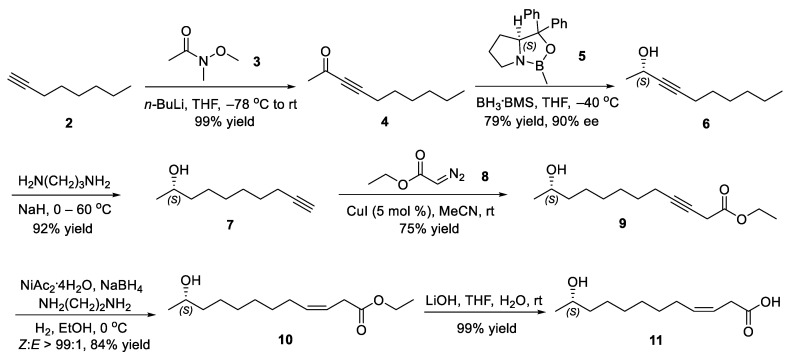
Synthesis of (*S*,*Z*)-11-hydroxydodec-3-enoic acid.

Our investigation commenced with the synthesis of (*S*,*Z*)-11-hydroxydodec-3-enoic acid (**11**), the key precursor of the target pheromone (Scheme 4). The reaction of Weinreb’s amide **3** with an alkynyllithium, prepared *in situ* from oct-1-yne (**2**) and *n*-butyllithium, afforded dec-3-yn-2-one (**4**) in a near quantitative yield (99%) [21,22]. The construction of the crucial stereocenter was then achieved through a catalytic enantioselective CBS (Corey–Bakshi–Shibata) reduction [23,24]. Employing oxazaborolidine **5** and the complex of borane-dimethyl sulfide converted ynone **4** to (*S*)-dec-3-yn-2-ol (**6**) (79% yield, 90% ee, determined by chiral HPLC of its 3,5-dinitrobenzoate; see Supplementary Materials for details). Moreover, the specific rotation of **6** (${\left[\alpha \right]}_{D}^{25}$ = −22.05 (c = 2.54, CHCl_3_) was consistent with the data of the literature [25] (${\left[\alpha \right]}_{D}^{22}$ = −23.7 (c = 10.2), the enantiomeric purity). The subsequent zipper reaction relocated the triple bond of chiral propargylic alcohol **6** to the terminal position, delivering (*S*)-dec-9-yn-2-ol (**7**) in 92% yield [26,27,28,29]. A pivotal carbon–carbon bond formation developed by Fu and co-workers was realized through a copper(I)-catalyzed coupling of terminal alkyne **7** with ethyl diazoacetate (**8**) to furnish ethyl (*S*)-11-hydroxydodec-3-ynoate (**9**) [30]. The stereoselective partial hydrogenation of alkynyl ester **9** catalyzed with Ni(OAc)_2_/NaBH_4_ provided the corresponding *cis* alkenyl ester **10** (84% yield, *Z*:*E* > 99:1, determined by ^13^C NMR spectra) [31,32,33,34]. Finally, the saponification of ethyl (*S*,*Z*)-11-hydroxydodec-3-enoate (**10**) with lithium hydroxide proceeded successfully to yield hydroxyl acid **11** almost quantitatively [35].

### 2.3. Synthesis of Ferrulactone II (S)-1 and Its Enantiomer (R)-1

**Scheme 5:**
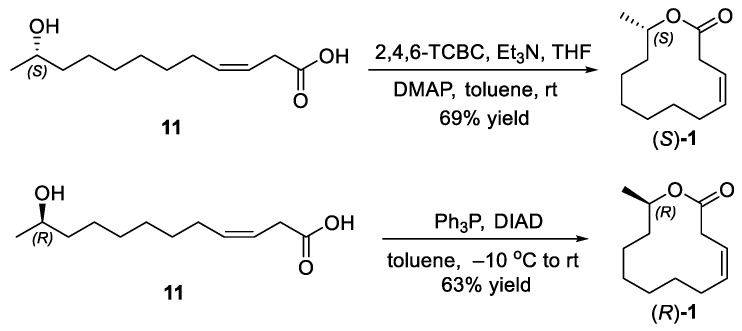
Synthesis of Ferrulactone II and its enantiomer.

With the key precursor (*S*,*Z*)-11-hydroxydodec-3-enoic acid (**11**) in hand, both enantiomers of the target macrolactone (*S*)- and (*R*)-**1** were synthesized (Scheme 5). Ferrulactone II ((*S*)-**1**) was prepared firstly using Yamaguchi esterification, which has advantages of mild conditions and high efficiency [36,37]. Treatment of hydroxy acid **11** with 2,4,6-trichlorobenzoyl chloride and triethylamine in THF generated the mixed anhydride *in situ*, and the subsequent DMAP-catalyzed cyclization in toluene afforded (*S*,*Z*)-3-dodecen-11-olide ((*S*)-**1**) [38]. Mitsunobu-macrolactonization could invert the configuration of the chiral carbon; therefore, we choose this strategy to prepare the enantiomer of Ferrulactone II [39,40]. The reaction of hydroxy acid **11** with triphenylphosphine and DEAD in toluene provided (*R*,*Z*)-3-dodecen-11-olide ((*R*)-**1**). The specific rotations of both compounds, (*R*)-**1** and (*S*)-**1**, were in agreement with those described in the literature [17,20]. To further corroborate the absolute configuration of the target pheromone, the experimental ECD spectra were compared with the calculated ECD spectra [41,42]. The close match observed for both (*S*)-**1** and (*R*)-**1** (Figure S25) confirmed their absolute stereochemistry.

## 3. Materials and Methods

### 3.1. General Information

The details of the instruments and experimental methods are described in the Supporting Information.

### 3.2. Synthesis of Dec-3-yn-2-one (***4***) (CAS 91658-50-3)

Under an argon atmosphere, a 250 mL Schlenk flask containing a stir bar was charged with 1-octyne (**2**) (5.51 g, 50.00 mmol) and anhydrous THF (100 mL) at room temperature. The solution was cooled to −78 °C, then *n*-BuLi (21 mL, 2.5 M in *n*-hexane, 52.50 mmol) was added dropwise. The resulting mixture was stirred for 2 h at the same temperature, and *N*-methoxy-*N*-methylacetamide (**3**) (5.67 g, 55.00 mmol) was added slowly. After the reaction mixture was warmed to room temperature and stirred for an additional 7 h, the reaction was quenched with saturated aqueous NH_4_Cl (30 mL). The organic layer was separated, and the aqueous phase was back-extracted with Et_2_O (3 × 30 mL). The combined organic layers were washed with brine (90 mL), dried over anhydrous Na_2_SO_4_, filtered, and concentrated under reduced pressure. The final purification by silica-gel column chromatography (petroleum ether/EtOAc 100:1) afforded dec-3-yn-2-one (**4**) (7.60 g, 99% yield) as a pale-yellow oil. ^1^H NMR (500 MHz, CDCl_3_) δ 2.28 (t, *J* = 7.1 Hz, 2H), 2.25 (s, 3H), 1.53–1.48 (m, 2H), 1.36–1.30 (m, 2H), 1.27–1.20 (m, 4H), 0.83 (t, *J* = 6.8 Hz, 3H). ^13^C NMR (126 MHz, CDCl_3_) δ 185.1, 94.4, 81.6, 32.9, 31.4, 28.7, 27.8, 22.6, 19.1, 14.2. HRMS (ESI): calculated for C_10_H_17_O [M + H]^+^: 153.1274, found: 153.1278 (Δ = 2.4 ppm).

### 3.3. Synthesis of (S)-Dec-3-yn-2-ol (***6***) (CAS 72132-11-7)

A 250 mL Schlenk flask containing a stir bar and freshly activated 4 Å molecular sieves was evacuated and back-filled with argon three times at room temperature. After being cooled to −40 °C, oxazaborolidine (**5**) (12 mL, 1 M in toluene, 12.00 mmol) and THF (10 mL) were added. BH_3_·BMS (12 mL, 2 M in THF, 24.00 mmol) was then added dropwise, and the mixture was stirred for 2 h. Subsequently, a solution of dec-3-yn-2-one (**4**) (3.04 g, 20.00 mmol) in THF (50 mL) was added via a syringe pump over 48 h. The reaction mixture was stirred for an additional 7 h at −40 °C, and quenched with MeOH (20 mL) at the same temperature. The resulting mixture was warmed to room temperature and stirred for 3 h. The mixture was filtered, and the filtrate was concentrated under reduced pressure. The final purification by silica-gel column chromatography (petroleum ether/EtOAc 20:1) gave (*S*)-dec-3-yn-2-ol (**6**) (2.43 g, 79% yield, 90% ee, determined by chiral HPLC of its 3,5-dinitrobenzoate) as a colorless oil. ${\left[\alpha \right]}_{D}^{25}$ = −22.05 (c = 2.54, CHCl_3_). Lit [25]. ${\left[\alpha \right]}_{D}^{22}$ = −23.7 (c = 10.2). ^1^H NMR (500 MHz, CDCl_3_) δ 4.54–4.49 (m 1H), 2.19 (td, *J* = 7.2, 2.0 Hz, 2H), 1.74 (d, *J* = 4.8 Hz, 1H), 1.51–1.47 (m, 2H), 1.43 (d, *J* = 6.4 Hz, 3H), 1.40–1.36 (m, 2H), 1.32–1.25 (m, 4H), 0.89 (t, *J* = 6.9 Hz, 3H). ^13^C NMR (126 MHz, CDCl_3_) δ 84.9, 82.3, 58.8, 31.5, 28.8, 28.76, 24.9, 22.7, 18.8, 14.2. HRMS (ESI): calculated for C_10_H_19_O [M + H]^+^: 155.1431, found: 155.1431 (Δ = 0.0 ppm).

### 3.4. Synthesis of (S)-Dec-9-yn-2-ol (***7***) (CAS 107351-67-7)

A 100 mL three-necked flask containing a stir bar was charged with freshly activated 4 Å molecular sieves (2.00 g) and NaH (3.46 g, 144.00 mmol, 60% dispersion in mineral oil) at room temperature. 1,3-Propanediamine (25 mL) was added, and the resulting suspension was heated to 60 °C and refluxed for 5 h (caution: gas evolution was observed). Subsequently, the suspension was cooled to 0 °C, and (*S*)-dec-3-yn-2-ol (**6**) (2.22 g, 14.40 mmol) was then added. After the reaction mixture was warmed to room temperature and stirred for 4 h, it was quenched with ice water (10 mL) at 0 °C. The mixture was extracted with Et_2_O (3 × 30 mL), and the combined organic layers were washed with brine (90 mL), dried over Na_2_SO_4_, filtered, and concentrated under reduced pressure. The final purification by silica-gel column chromatography (petroleum ether/EtOAc 20:1) afforded (*S*)-dec-9-yn-2-ol (**7**) (2.05 g, 92% yield) as a pale-yellow oil. ${\left[\alpha \right]}_{D}^{25}$ = +9.22 (c = 3.47, CHCl_3_) [43]. ${\left[\alpha \right]}_{D}^{21.2}$ = +9.4 (c = 1.02, CHCl_3_). ^1^H NMR (500 MHz, CDCl_3_) δ 3.81–3.76 (m, 1H), 2.19 (td, *J* = 7.1, 2.6 Hz, 2H), 1.94 (t, *J* = 2.7 Hz, 1H), 1.56–1.50 (m, 2H), 1.45–1.31 (m, 8H), 1.19 (d, *J* = 6.2 Hz, 3H). ^13^C NMR (126 MHz, CDCl_3_) δ 84.8, 68.3, 68.3, 39.4, 29.2, 28.8, 28.5, 25.8, 23.7, 18.5. HRMS (ESI): calculated for C_10_H_19_O [M + H]^+^: 155.1430, found: 155.1437 (Δ = 0.64 ppm).

### 3.5. Synthesis of Ethyl (S)-11-Hydroxydodec-3-ynoate (***9***) (New Compound)

Under an argon atmosphere, a 50 mL Schlenk tube containing a stir bar was charged with CuI (57 mg, 0.30 mmol) and anhydrous MeCN (10 mL) at room temperature. (*S*)-Dec-9-yn-2-ol (**7**) (0.93 g, 6.00 mmol) and ethyl diazoacetate (**8**) (0.82 g, 7.20 mmol) were then added. The reaction mixture was stirred at room temperature for 17 h, filtered, and concentrated under reduced pressure. The final purification by silica-gel column chromatography (petroleum ether/EtOAc, 50:1) afforded ethyl (*S*)-11-hydroxydodec-3-ynoate (**9**) (1.08 g, 75% yield) as a colorless oil. ${\left[\alpha \right]}_{D}^{25}$ = +12.67 (c = 2.21, CHCl_3_). ^1^H NMR (500 MHz, CDCl_3_) δ 4.19 (q, *J* = 7.1 Hz, 2H), 3.81–3.76 (m, 1H), 3.24 (t, *J* = 2.5 Hz, 2H), 2.21–2.18 (m, 2H), 1.60 (br s, 1H), 1.52–1.48 (m, 2H), 1.45–1.36 (m, 6H), 1.33–1.31 (m, 2H), 1.28 (t, *J* = 5.3 Hz, 3H), 1.19 (d, *J* = 6.1 Hz, 3H). ^13^C NMR (126 MHz, CDCl_3_) δ 169.2, 83.9, 71.7, 68.3, 61.6, 39.4, 29.2, 28.9, 28.7, 26.3, 25.7, 23.6, 18.9, 14.3. HRMS (ESI): calculated for C_14_H_25_O_3_ [M + H]^+^: 241.1798, found: 241.1800 (Δ = 0.66 ppm).

### 3.6. Synthesis of Ethyl (S,Z)-11-Hydroxydodec-3-enoate (***10***) (CAS 3054699-79-2)

A 50 mL Schlenk tube containing a stir bar and Ni(OAc)_2_·4H_2_O (120 mg, 0.48 mmol) was evacuated and back-filled with H_2_ three times. NaBH_4_ (18 mg, 0.48 mmol) in anhydrous EtOH (10 mL) was then added slowly (caution: gas evolution was observed). After stirring for 1 h at room temperature, the mixture was cooled to 0 °C. Ethylenediamine (115 mg, 1.91 mmol) and a solution of ethyl (*S*)-11-hydroxydodec-3-ynoate (**9**) (460 mg, 1.91 mmol) in anhydrous EtOH (1 mL) were added sequentially. Under a H_2_ atmosphere, the reaction mixture was stirred at 0 °C for 1 h, filtered, and concentrated under reduced pressure. The final purification by silica-gel chromatography (petroleum ether/EtOAc 50:1) afforded ethyl (*S,Z*)-11-hydroxydodec-3-enoate (**10**) (392 mg, 84% yield, *Z*:*E* > 99:1, determined by ^13^C NMR spectra) as a colorless oil. ${\left[\alpha \right]}_{D}^{25}$ = +22.46 (c = 2.85, CHCl_3_). ^1^H NMR (500 MHz, CDCl_3_) δ 5.60–5.53 (m, 2H), 4.14 (q, *J* = 7.0 Hz, 2H), 3.81–3.76 (m, 1H), 3.07 (d, *J* = 6.0 Hz, 2H), 2.06–2.02 (m, 2H), 1.66 (br s, 1H), 1.45–1.29 (m, 10H), 1.26 (t, *J* = 7.0 Hz, 3H), 1.19 (d, *J* = 6.2 Hz, 3H). ^13^C NMR (126 MHz, CDCl_3_) δ 172.2, 133.5, 121.0, 68.3, 60.4, 39.5, 33.2, 29.6, 29.4, 29.3, 27.5, 25.8, 23.6, 14.3. HRMS (ESI): calculated for C_14_H_27_O_3_ [M + H]^+^: 243.1955, found: 243.1952 (Δ = −1.0 ppm).

### 3.7. Synthesis of (S,Z)-11-Hydroxydodec-3-enoic Acid (***11***) (CAS 87583-43-5)

Under an argon atmosphere, a 50 mL Schlenk tube containing a stir bar was charged with ethyl (*S,Z*)-11-hydroxydodec-3-enoate (**10**) (390 mg, 1.61 mmol), THF (9 mL), and H_2_O (3 mL) at room temperature. LiOH (1.2 mL, 2 M in H_2_O, 2.40 mmol) was then added. After the reaction mixture was stirred for 24 h at room temperature, it was acidified to pH 2 with 1 M HCl. The resulting mixture was extracted with EtOAc (3 × 30 mL), and the combined extracts were dried over Na_2_SO_4_, filtered, and concentrated under reduced pressure. The final purification by silica-gel column chromatography (petroleum ether/EtOAc 2:1) afforded (*S*,*Z*)-11-hydroxydodec-3-enoic acid (**11**) (344 mg, 99% yield) as a colorless oil. ${\left[\alpha \right]}_{D}^{25}$ = +21.05 (c = 2.09, CHCl_3_). ^1^H NMR (500 MHz, CDCl_3_) δ 5.64–5.52 (m, 2H), 3.84–3.78 (m, 1H), 3.13 (d, *J* = 6.4 Hz, 2H), 2.07–2.03 (m, 2H), 1.44–1.36 (m, 4H), 1.32–1.25 (m, 6H), 1.19 (d, *J* = 6.2 Hz, 3H). ^13^C NMR (126 MHz, CDCl_3_) δ 176.9, 134.2, 120.4, 68.4, 39.3, 32.7, 29.4, 29.1, 29.1, 27.4, 25.7, 23.6. HRMS (ESI): calculated for C_12_H_23_O_3_ [M + H]^+^: 215.1642, found: 215.1641 (Δ = −0.28 ppm).

### 3.8. Synthesis of (S,Z)-12-Methyloxacyclododec-4-en-2-one ((S)-***1***) (CAS 86578-99-6)

Under an argon atmosphere, a 10 mL Schlenk tube containing a stir bar was charged with (*S,Z*)-11-hydroxydodec-3-enoic acid (**11**) (37 mg, 0.17 mmol) and THF (3 mL) at room temperature. Et_3_N (86 mg, 0.85 mmol) was then added, followed by 2,4,6-trichlorobenzoyl chloride (63 mg, 0.26 mmol). After the reaction mixture was stirred for 1 h at room temperature, it was filtered and concentrated under reduced pressure to afford the crude 2,4,6-trichlorobenzoic (*S*,*Z*)-11-hydroxydodec-3-enoic anhydride.

A 500 mL three-necked flask containing a stir bar and DMAP (415 mg, 3.40 mmol) was evacuated and back-filled with argon three times. Anhydrous toluene (250 mL) was added, and the resulting solution was stirred vigorously. A solution of the above crude anhydride in toluene (40 mL) was then added dropwise via a syringe pump over 2 h. After the reaction mixture was stirred for an additional 16 h at room temperature, it was filtered and concentrated under reduced pressure. The final purification by silica-gel column chromatography (petroleum ether/EtOAc 20:1) afforded (*3Z*,*11S*)-3-dodecen-11-olide ((*S*)-**1**) (23 mg, 69% yield) as a colorless oil. ${\left[\alpha \right]}_{D}^{25}$ = +56.08 (c = 1.03, CHCl_3_). Lit [17]. ${\left[\alpha \right]}_{D}^{22.5}$ = +70.5 (c = 0.96, CHCl_3_). ^1^H NMR (500 MHz, CDCl_3_) δ 5.59–5.52 (m, 2H), 4.94–4.88 (m, 1H), 3.09–2.98 (m, 2H), 2.07–2.02 (m, 2H), 1.55–1.48 (m, 2H), 1.31–1.25 (m, 8H), 1.20 (d, *J* = 6.3 Hz, 3H). ^13^C NMR (126 MHz, CDCl_3_) δ 171.7, 133.8, 121.3, 71.2, 36.1, 33.8, 29.5, 29.2, 29.2, 27.4, 25.4, 20.3. HRMS (ESI): calculated for C_12_H_21_O_2_ [M + H]^+^: 197.1536, found: 197.1536 (Δ = 0.0 ppm).

### 3.9. Synthesis of (R,Z)-12-Methyloxacyclododec-4-en-2-one ((R)-***1***) (CAS 87583-38-8)

A 25 mL Schlenk tube containing a stir bar and triphenylphosphine (262 mg, 1.00 mmol) was vacuumed and back-filled with argon three times at room temperature. Then, a solution of hydroxy acid (**11**) (30 mg, 0.14 mmol) in toluene (15 mL) was added and cooled to −10 °C. DIAD (98 mg, 0.56 mmol) was added dropwise over 10 min, and the resulting mixture was warmed to room temperature and stirred for 2 h. The reaction mixture was filtered, and the filtrate was concentrated under reduced pressure. The final purification by silica gel column chromatography (petroleum ether/EtOAc 20:1) afforded (*R,Z*)-12-methyloxacyclododec-4-en-2-one ((*R*)-**1**) (17 mg, 63% yield) as a colorless oil. ${\left[\alpha \right]}_{D}^{25}$ = −66.17 (c = 1.07, CHCl_3_). Lit [20]. ${\left[\alpha \right]}_{D}^{22}$ = −68.1 (c = 0.7, CHCl_3_) ^1^H NMR (500 MHz, CHCl_3_) δ 5.60–5.52 (m, 2H), 4.94–4.89 (m, 1H), 3.10–2.98 (m, 2H), 2.07–2.03 (m, 2H), 1.55–1.49 (m, 2H), 1.29–1.23 (m, 8H), 1.20 (d, *J* = 6.3 Hz, 3H) ^13^C NMR (126 MHz, CHCl_3_) δ 171.8, 133.8, 121.3, 71.2, 36.1, 33.7, 29.9, 29.4, 29.1, 27.4, 25.3, 20.3. HRMS (ESI): calculated for C_12_H_21_O_2_ [M + H]^+^: 197.1536, found: 197.1539 (Δ = 1.2 ppm).

## 4. Conclusions

In summary, an efficient and concise asymmetric synthesis of Ferrulactone II, the aggregation pheromone of *Cryptolestes ferrugineus*, and its enantiomer, has been developed. Compared to previous syntheses, this approach provides a concise and protecting-group-free route with high overall yield, enabling access to both enantiomers using a single chiral catalyst. Ferrulactone II was obtained in seven steps with an overall yield of 31%, while its enantiomer was prepared in seven steps with a total yield of 28%. All synthetic intermediates and final products were fully characterized by ^1^H and ^13^C NMR spectroscopy and HRMS (Figures S1–S22). Importantly, this synthetic approach provides reliable access to both enantiomers of Ferrulactone II, supplying well-defined material suitable for future biological evaluation and studies aimed at exploring their potential application in the management of the rusty grain beetle.

## Data Availability

The original contributions presented in this study are included in the article/Supplementary Materials. Further inquiries can be directed to the corresponding author.

## Supplementary Materials

The following supporting information can be downloaded at: https://www.mdpi.com/article/10.3390/molecules31030404/s1. Research on the optical purity of chiral alcohol **6**, ^1^H NMR and ^13^C NMR spectra for all the synthetic compounds, and HPLC chromatography of the 3,5-dinitrobenzoate of **6** and ECD spectra of the target compounds (*S*)- and (*R*)-**1**. Refs. [44,45,46] are cited in the Supplementary Materials.
