# Supplementary material for: A Concise Asymmetric Synthesis of the Aggregation Pheromone of Cryptolestes ferrugineus, Ferrulactone II, and Its Enantiomer
Source: Molecules. 2026 Jan 24;31(3):404. doi: 10.3390/molecules31030404 (PMC12898138; doi:10.3390/molecules31030404)
Supplement: Supplementary file 1 [file molecules-31-00404-s001.zip › molecules-4091757-supplementary.pdf]

SUPPORTING INFORMATION

A Concise Asymmetric Synthesis of the Aggregation Pheromone of *Cryptolestes ferrugineus*, Ferrulactone II, and Its Enantiomer

Hong Tang, Biyu An, Yiwen Huang, Dan Liu, Qinghua Bian, Jiangchun Zhong*

Department of Applied Chemistry, China Agricultural University, 2 West Yuanmingyuan Road, Beijing 100193, China

* Correspondence: zhong@cau.edu.cn

Table of Contents

1. General Information……………………………………………………………………….…….S2

2. The Research on the Optical Purity of Chiral Alcohol **6**………….……….……..…….…S2

3. ^{1}H, ^{13}C NMR Spectra of the Products……………………………………………………….S4

4. HPLC Chromatography and ECD spectra of the Compounds………………………….S15

5. References……………………………………………………………………………….S16

1. General Information

Unless otherwise noted, all reactions were performed under an argon atmosphere using standard Schlenk techniques with magnetic stirring. All commercial reagents and solvents were used as received, and toluene was dried over calcium hydride (CaH_2) and freshly distilled prior to use. Enantiomeric excesses (ee) was determined by an Agilent 1200 HPLC with a Chiralpak AD-H column. Optical rotations were measured on a Rudolph AUTOPOL-IV polarimeter, with a 0.25 dm cell length. ^1H and ^{13}C NMR spectra were recorded on a Bruker AscendTM 500 MHz spectrometer and using deuterated solvent. Chemical shifts were reported in parts per million (ppm) with an internal standard of tetramethylsilane (0.00 ppm) for ^1H NMR and the residual chloroform (77.16 ppm) in $CDCl_3$ for ^{13}C NMR. High-resolution mass spectra (HRMS) analyses were performed on Waters LCT Premier™ with an ESI mass spectrometer. The experimental ECD spectrum was obtained using a JASCO J-1500 spectropolarimeter equipped with a 1 mm path length quartz cell, while the theoretical ECD calculations were conducted at the DFT/B3LYP level using the Gaussian 09 software package.

2. The Research on the Optical Purity of Chiral Alcohol 6

Scheme S1. Synthesis of racemic alcohol *rac*-**6**.

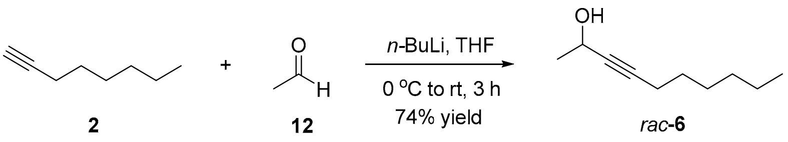

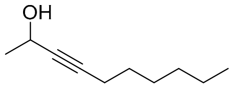

dec-3-yn-2-ol (*rac*-6) (CAS 69668-93-5)[1]

Under an argon atmosphere, a 50 mL Schlenk tube containing a stir bar was charged with 1-octyne (**2**) (0.55 g, 5.00 mmol) in anhydrous THF (5 mL) at 0 °C, a solution of *n*-BuLi (2.1 mL, 2.5 M in *n*-hexane, 5.25 mmol) was then added dropwise. The resulting mixture was stirred at the same temperature for 30 min, acetaldehyde (**12**) (0.26 g, 6.00 mmol) was added slowly. After the reaction mixture was warmed to room temperature and stirred for 3 h, the reaction was quenched with saturated aqueous NH_4Cl (5 mL). The organic layer was separated, and the aqueous phase was back-extracted with Et_2O (3 × 5 mL). The combined organic layers were washed with brine (15 mL), dried over anhydrous Na_2SO_4, filtered, and concentrated under reduced pressure. The final purification by silica-gel column chromatography (petroleum ether/EtOAc, 20:1) afforded dec-3-yn-2-ol (*rac*-**6**) (0.57 g, 74% yield) as a colorless oil. ^1H NMR (500 MHz, $CDCl_3$) δ 4.54 – 4.49 (m, 1H), 2.19 (td, *J* = 7.2, 2.0 Hz, 2H), 1.77 (d, *J* = 5.3 Hz, 1H), 1.52 – 1.47 (m, 2H), 1.43 (d, *J* = 6.5 Hz, 3H), 1.39 – 1.36 (m, 2H), 1.32 – 1.26 (m, 4H), 0.89 (t, *J* = 6.9 Hz, 3H). ^{13}C NMR (126 MHz, $CDCl_3$) δ 84.9, 82.3, 58.8, 31.5, 28.8, 28.7, 24.9, 22.7, 18.8, 14.2. HRMS(ESI): calculated for $C_{10}H_{19}O$ $[M+H]^+$: 155.1430, found: 155.1433 (Δ = 1.9 ppm).

Scheme S2. Synthesis of 3,5-dinitrobenzoates *rac*-**14** and (*S*)-**14**.

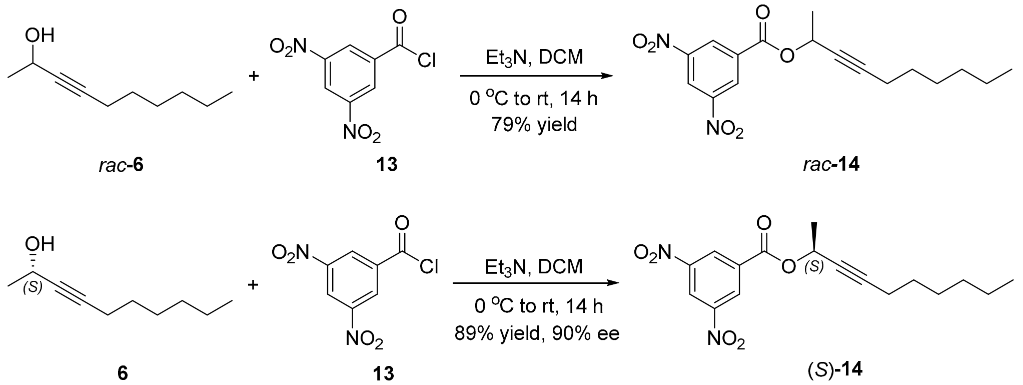

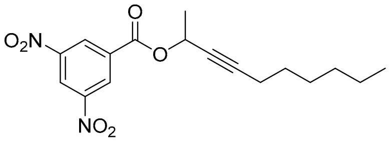

dec-3-yn-2-yl 3,5-dinitrobenzoate (*rac*-14) (New Compound)[2,3]

Under an argon atmosphere, a 50 mL Schlenk tube containing a stir bar was charged with dec-3-yn-2-ol (*rac*-**6**) (52 mg, 0.34 mmol) and dichloromethane (2 mL) at room temperature. The solution was cooled to 0 °C, triethylamine (76 mg, 0.75 mmol) and a solution of 3,5-dinitrobenzoyl chloride (**13**) (118 mg, 0.51 mmol) in dichloromethane (3 mL) were then added dropwise. After the reaction mixture was warmed to room temperature and stirred for 14 h, it was filtered, and concentrated under reduced pressure. The final purification by silica-gel column chromatography (petroleum ether/EtOAc, 50:1) afforded dec-3-yn-2-yl 3,5-dinitrobenzoate (*rac*-**14**) (93 mg, 79% yield) as a white solid. m.p. 64–66 °C. ^{1}H NMR (500 MHz, CDCl$_3$) δ 9.23 (t, *J* = 2.1 Hz, 1H), 9.18 (d, *J* = 2.2 Hz, 2H), 5.79 – 5.74 (m, 1H), 2.23 (td, *J* = 7.2, 1.9 Hz, 2H), 1.67 (d, *J* = 6.6 Hz, 3H), 1.54 – 1.51 (m, 2H), 1.39 – 1.36 (m, 2H), 1.31 – 1.27 (m, 4H), 0.88 (t, *J* = 6.8 Hz, 3H). ^{13}C NMR (126 MHz, CDCl$_3$) δ 161.7, 148.8, 134.1, 129.7, 122.6, 87.6, 77.6, 64.1, 31.4, 28.6, 28.4, 22.7, 22.0, 18.8, 14.1. HRMS (ESI): calculated for $C_{17}H_{20}O_6N_2Na$ [M+Na]$^+$: 377.1214, found: 377.1250 (Δ = 9.7 ppm).

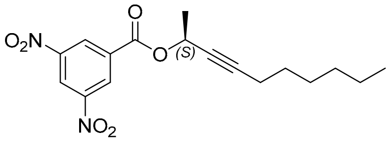

(*S*)-dec-3-yn-2-yl 3,5-dinitrobenzoate ((*S*)-14) (New Compound)

Following the similar procedure for 3,5-dinitrobenzoates *rac*-**14**, (*S*)-dec-3-yn-2-ol (**6**) (52 mg, 0.34 mmol) was converted to (*S*)-dec-3-yn-2-yl 3,5-dinitrobenzoate ((*S*)-**14**) (105 mg, 89% yield, 90% ee) as a white solid. The ee was determined by chiral HPLC (Daicel Chiralpak AD-H column, 3% isopropanol in *n*-hexane, 1.0 mL/min, 254 nm, minor t_r = 15.51 min (*R*), major t_r = 17.26 min (*S*)). m.p. 64–66 °C. $[\alpha]_D^{25}$ = −47.343 (c = 4.14, CHCl$_3$). ^{1}H NMR (500 MHz, CDCl$_3$) δ 9.23 (t, *J* = 2.2 Hz, 1H), 9.18 (d, *J* = 2.1 Hz, 2H), 5.78 – 5.74 (m, 1H), 2.23 (td, *J* = 7.2, 2.0 Hz, 2H), 1.67 (d, *J* = 6.7 Hz, 3H), 1.54 – 1.49 (m, 2H), 1.40 – 1.35 (m, 2H), 1.31 – 1.26 (m, 4H), 0.88 (t, *J* = 6.8 Hz, 3H). ^{13}C NMR (126 MHz, CDCl$_3$) δ 161.7, 148.8, 134.1, 129.7, 122.6, 87.6, 77.5, 64.1, 31.4, 28.6, 28.4, 22.7, 22.0, 18.8, 14.2. HRMS (ESI): calculated for $C_{17}H_{20}O_6N_2Na$ [M+Na]$^+$: 377.1214, found: 377.1249 (Δ = 9.3 ppm).

3. ^{1}H, ^{13}C NMR Spectra of the Products

Figure S1. ^{1}H NMR Spectrum of dec-3-yn-2-one (**4**) (500 MHz, $CDCl_3$).

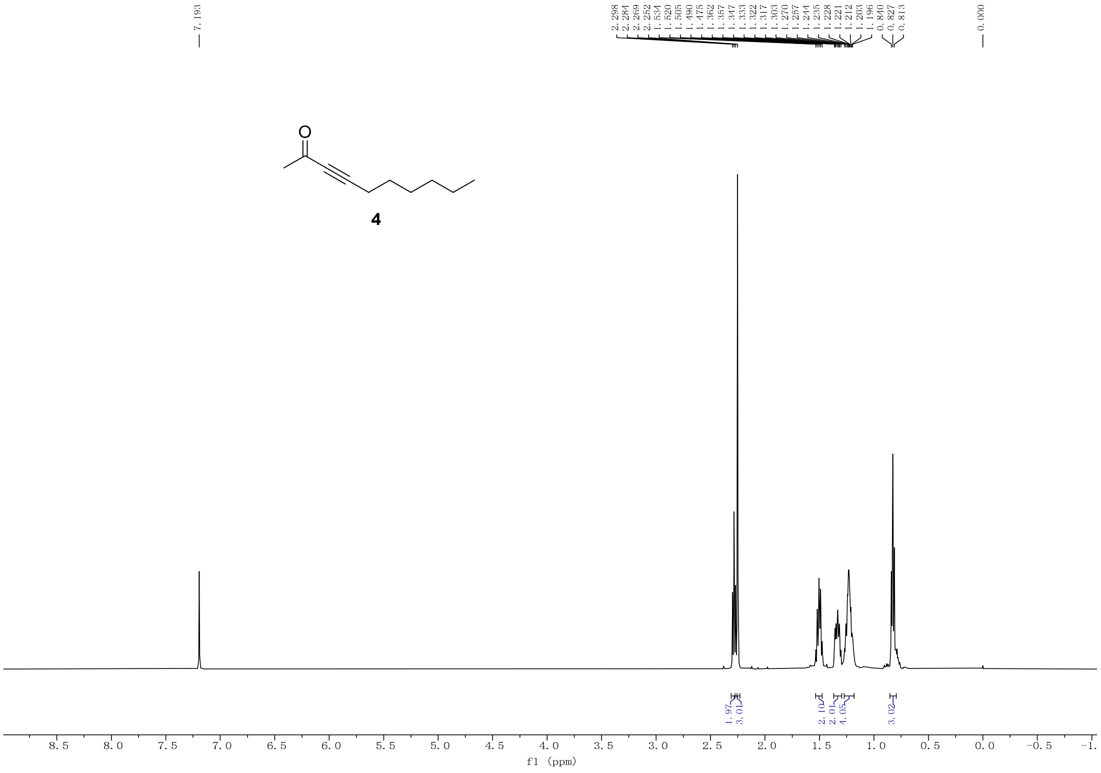

Figure S2. ^{13}C NMR Spectrum of dec-3-yn-2-one (**4**) (126 MHz, $CDCl_3$).

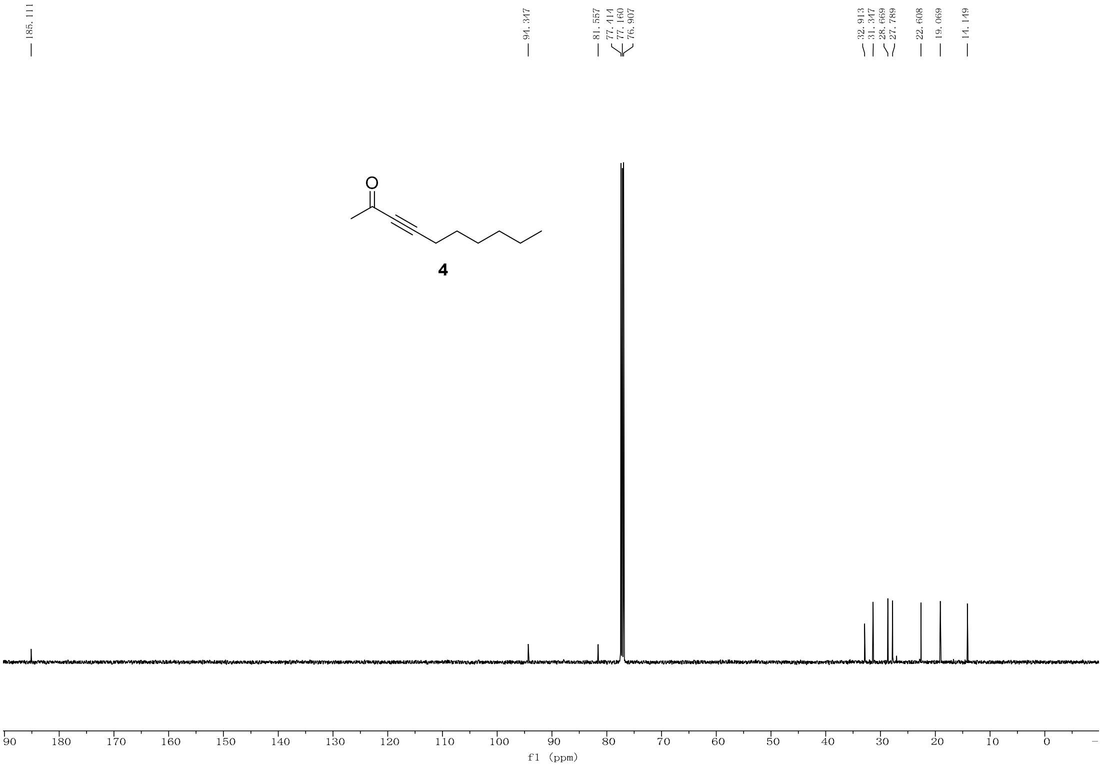

Figure S3. ^{1}H NMR Spectrum of (*S*)-dec-3-yn-2-ol (**6**) (500 MHz, $CDCl_3$).

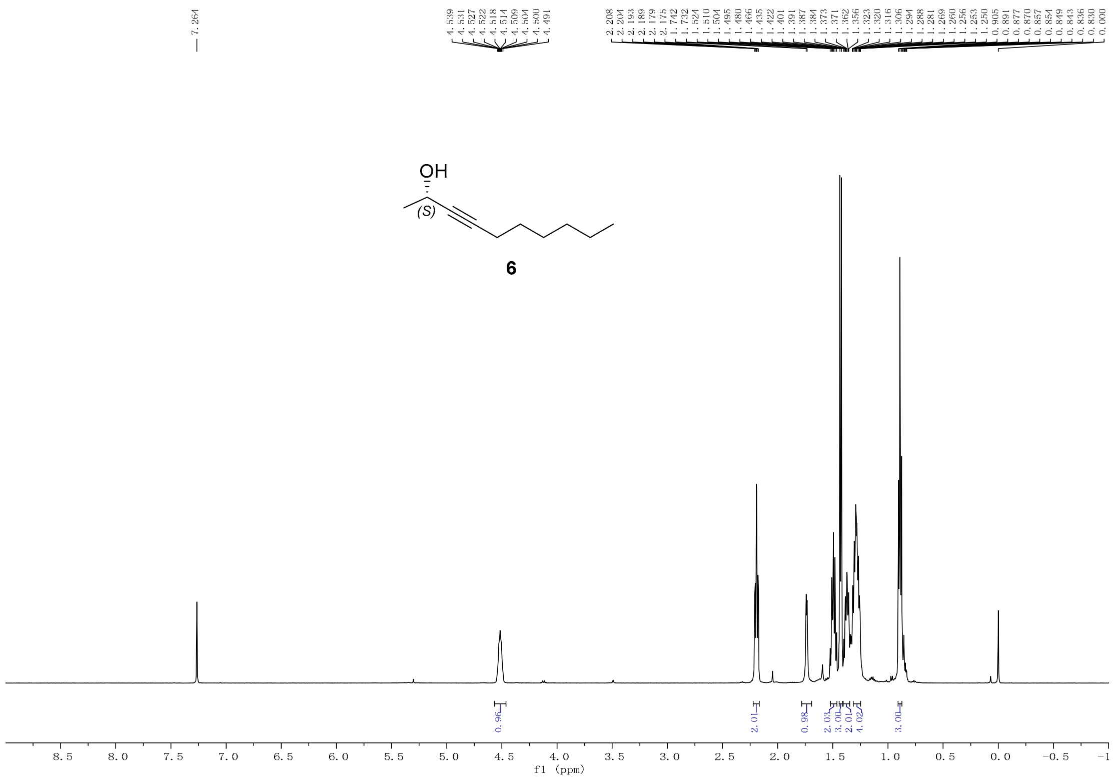

Figure S4. ^{13}C NMR (*S*)-dec-3-yn-2-ol (**6**) (126 MHz, $CDCl_3$).

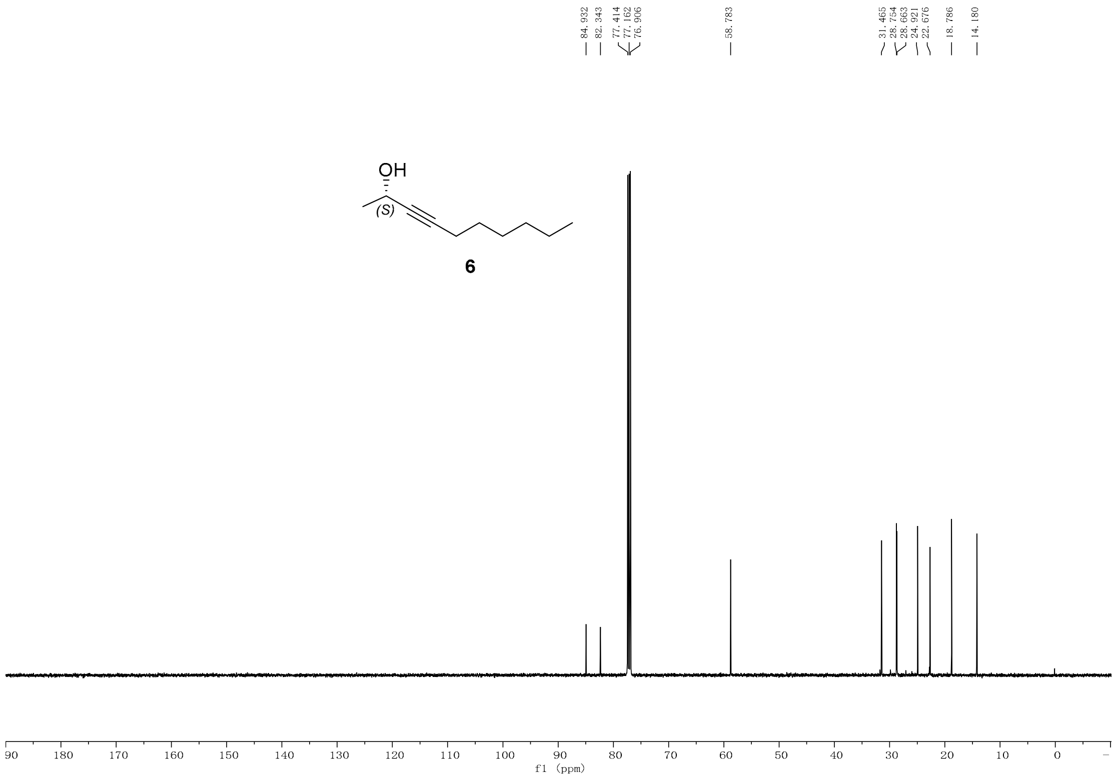

Figure S5. ^{1}H NMR Spectrum of (*S*)-dec-9-yn-2-ol (**7**) (500 MHz, $CDCl_3$).

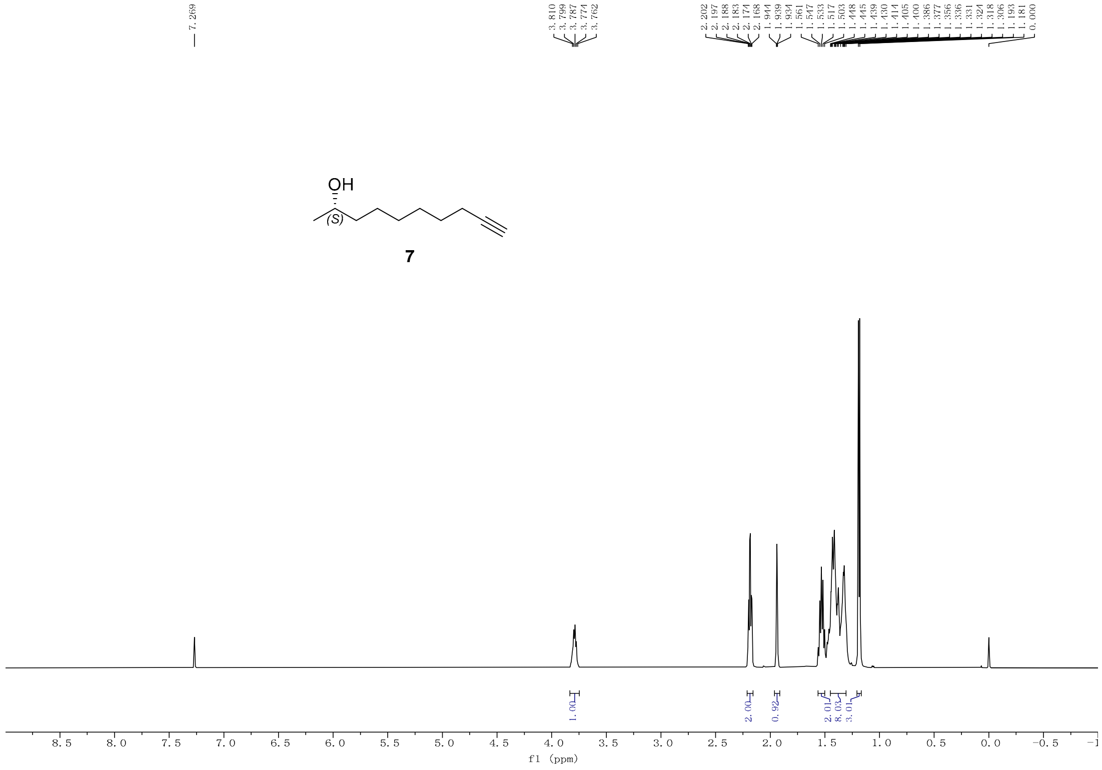

Figure S6. ^{13}C NMR Spectrum of (*S*)-dec-9-yn-2-ol (**7**) (126 MHz, $CDCl_3$).

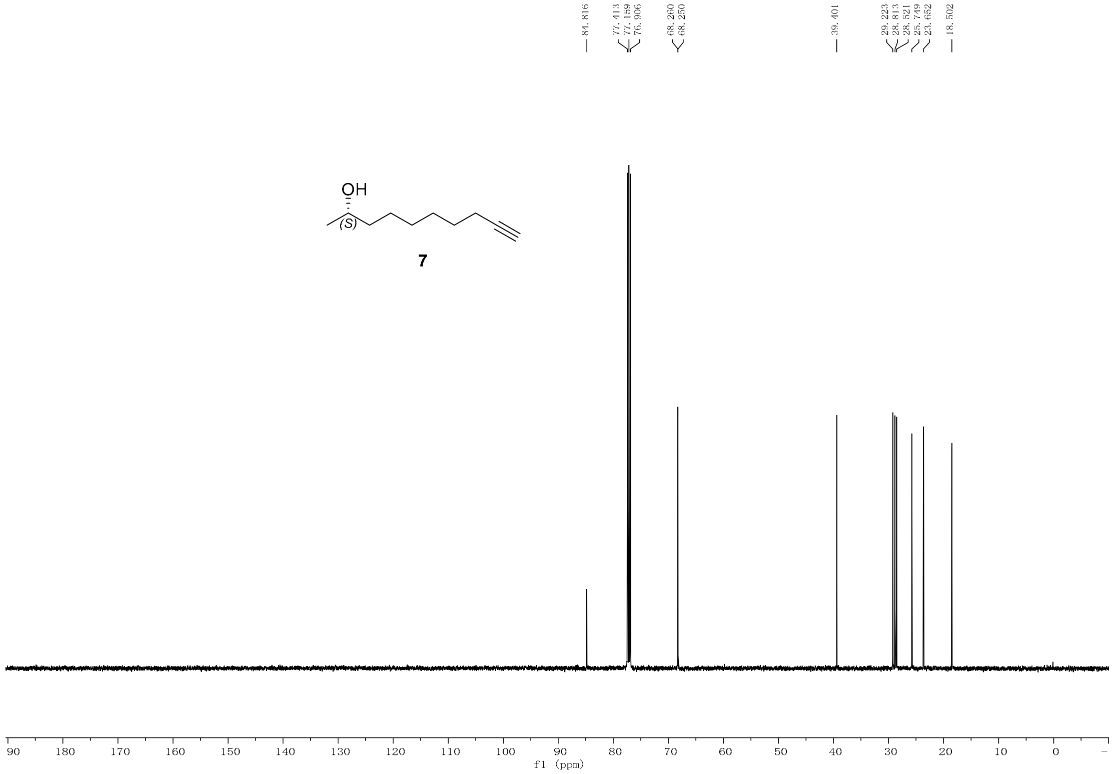

Figure S7. ^{1}H NMR Spectrum of ethyl (S)-11-hydroxydodec-3-ynoate ((**9**) (500 MHz, $CDCl_3$).

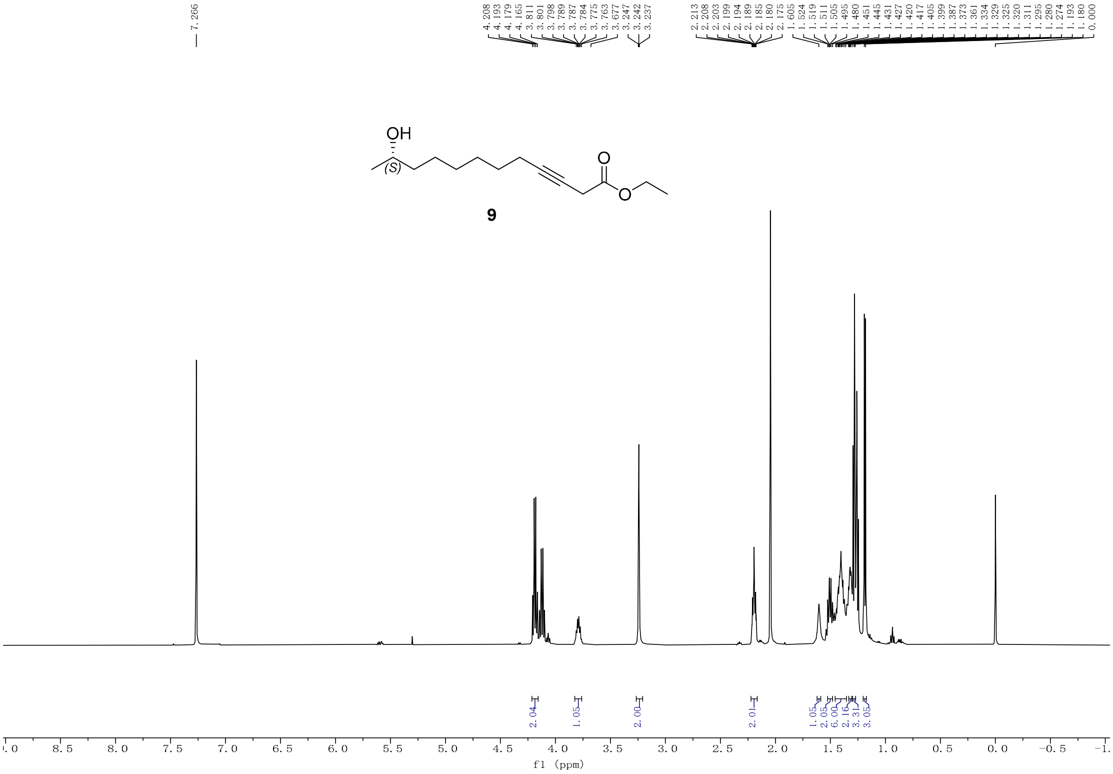

Figure S8. ^{13}C NMR Spectrum of ethyl (S)-11-hydroxydodec-3-ynoate ((**9**) (126 MHz, $CDCl_3$).

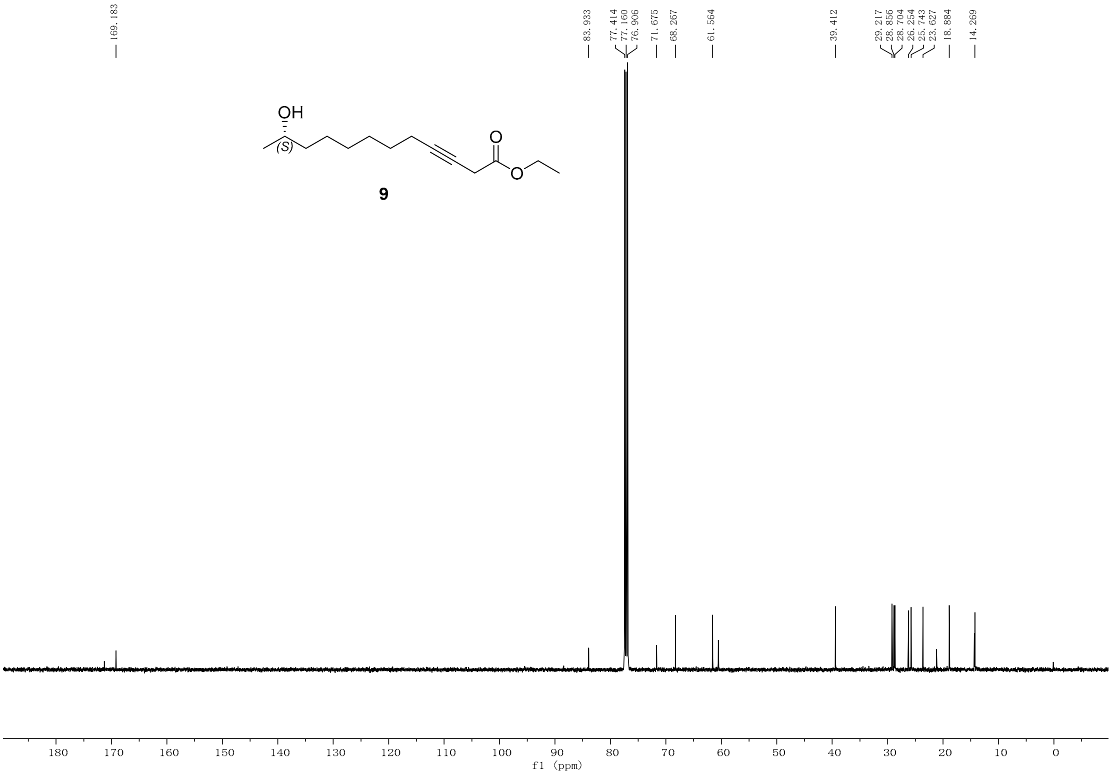

Figure S9. ^{1}H NMR Spectrum of ethyl (*S*,*Z*)-11-hydroxydodec-3-enoate (**10**) (500 MHz, $CDCl_3$).

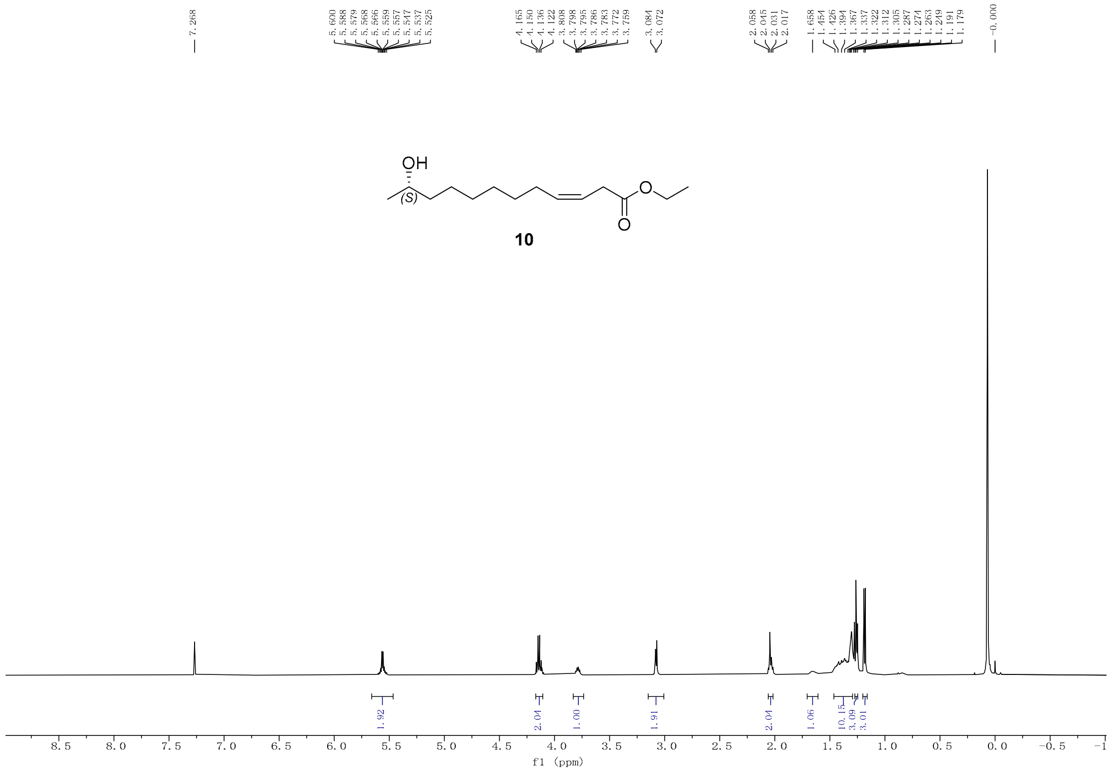

Figure S10. ^{13}C NMR Spectrum of (*S*,*Z*)-11-hydroxydodec-3-enoate (**10**) (126 MHz, $CDCl_3$).

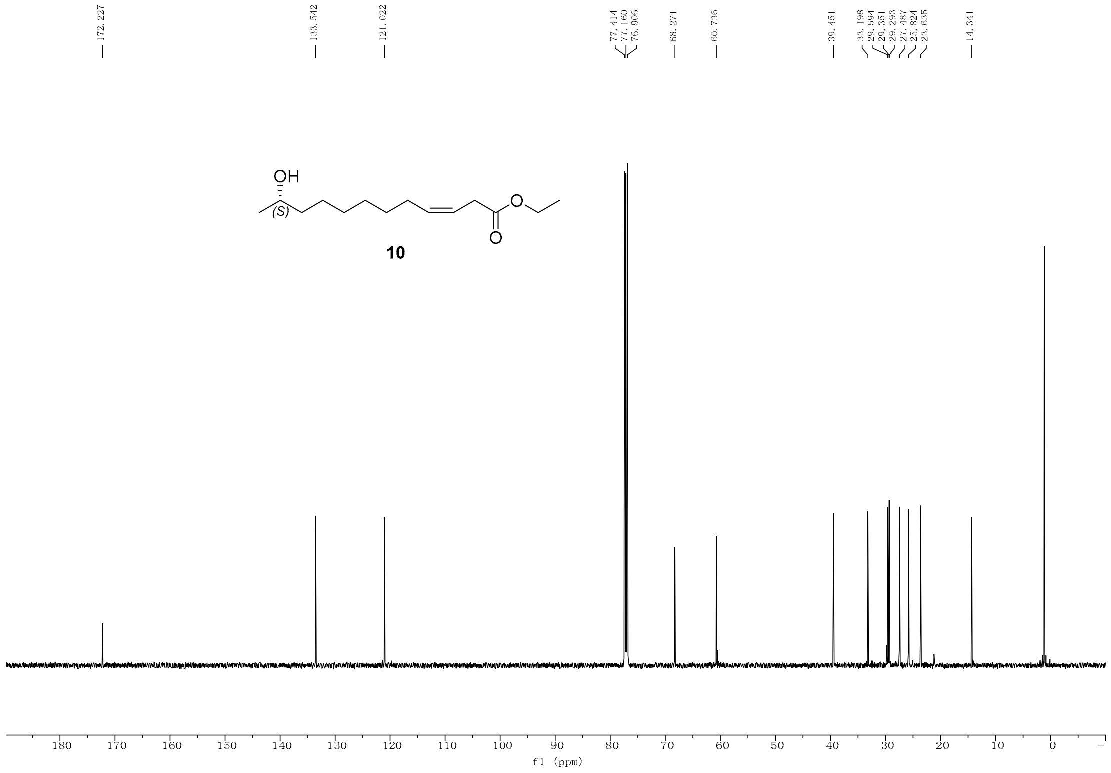

Figure S11. ^{1}H NMR Spectrum of (*S*,*Z*)-11-hydroxydodec-3-enoic acid (**11**) (500 MHz, $CDCl_3$).

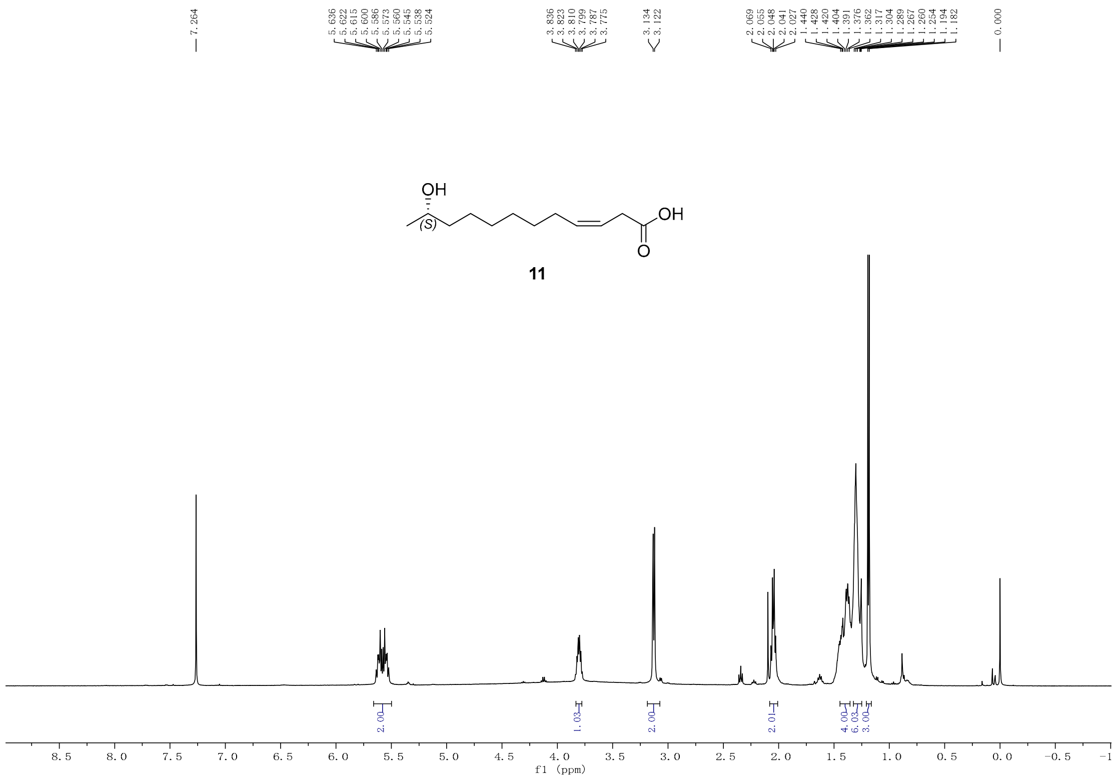

Figure S12. ^{13}C NMR Spectrum of (*S*,*Z*)-11-hydroxydodec-3-enoic acid (**11**) (126 MHz, $CDCl_3$).

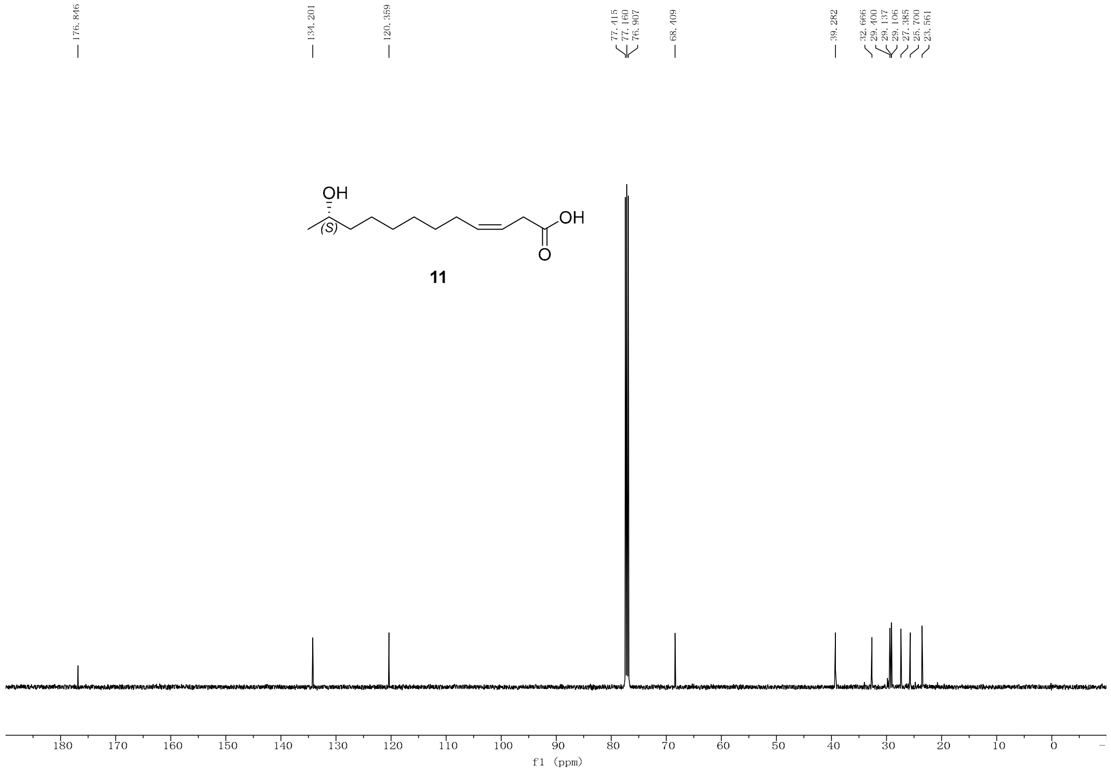

Figure S13. ^{1}H NMR Spectrum of (*S*,*Z*)-12-methyloxacyclododec-4-en-2-one ((*S*)-**1**) (500 MHz, $CDCl_3$).

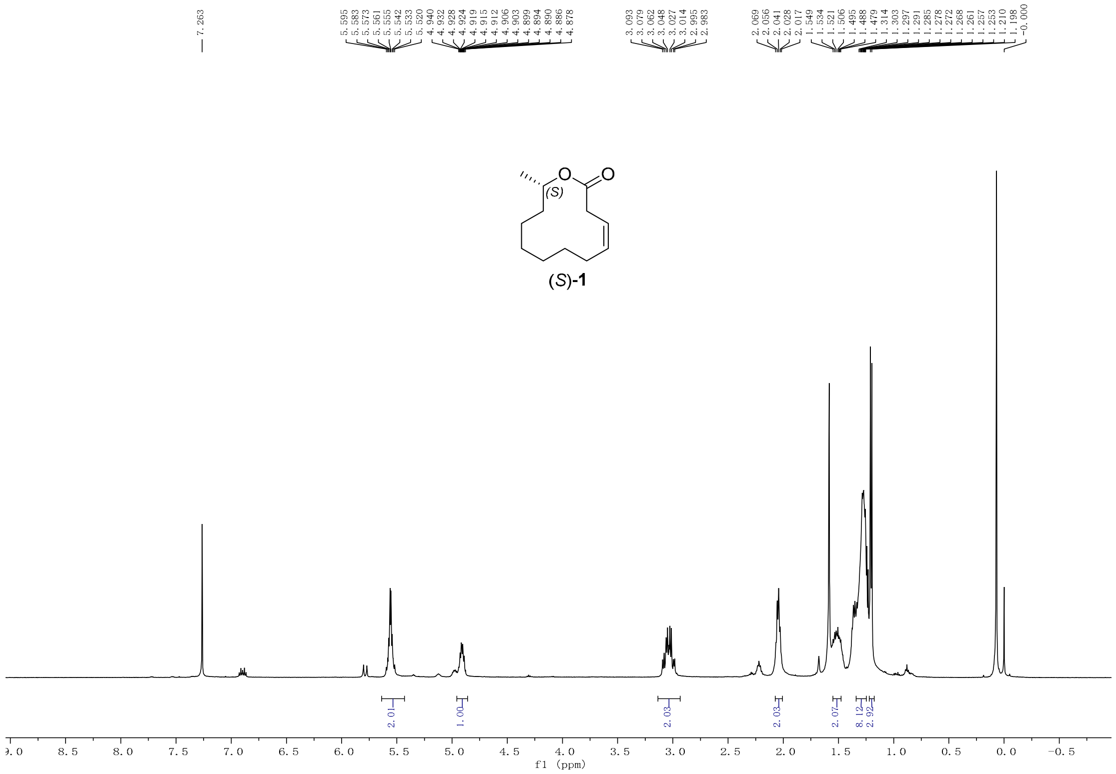

Figure S14. ^{13}C NMR Spectrum of (*S*,*Z*)-12-methyloxacyclododec-4-en-2-one ((*S*)-**1**) (126 MHz, $CDCl_3$).

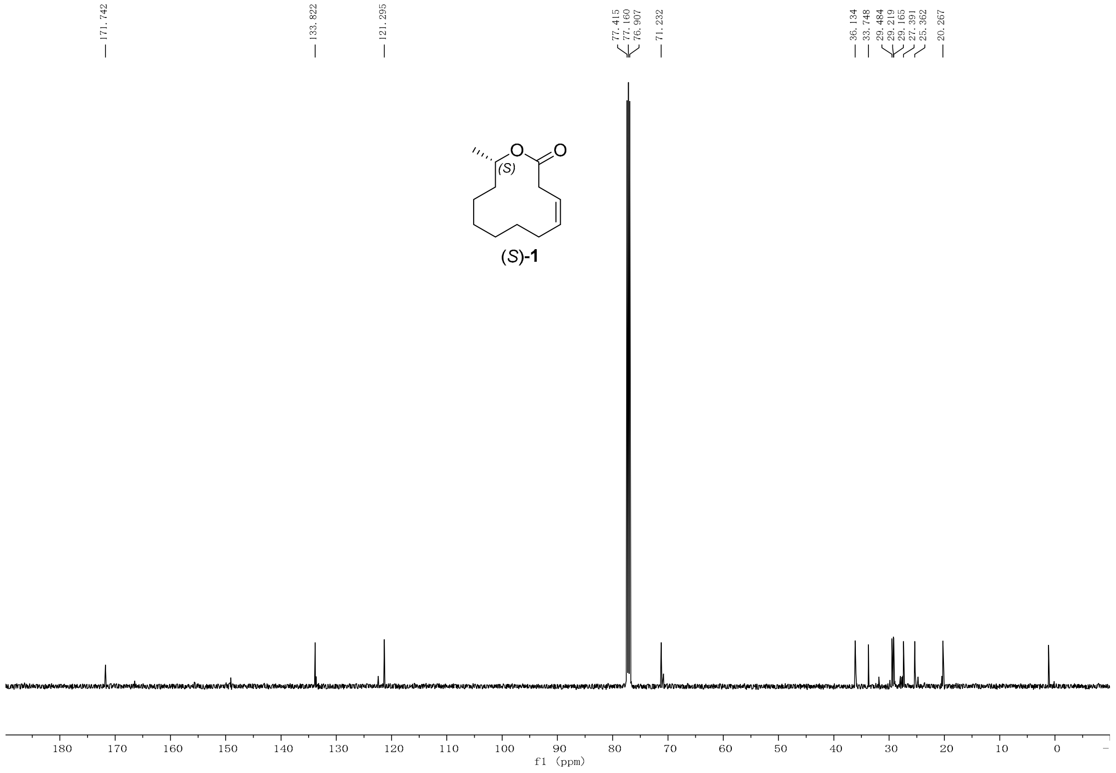

Figure S15. ^{1}H NMR Spectrum of (*R*,*Z*)-12-methyloxacyclododec-4-en-2-one ((*R*)-**1**) (500 MHz, $CDCl_3$).

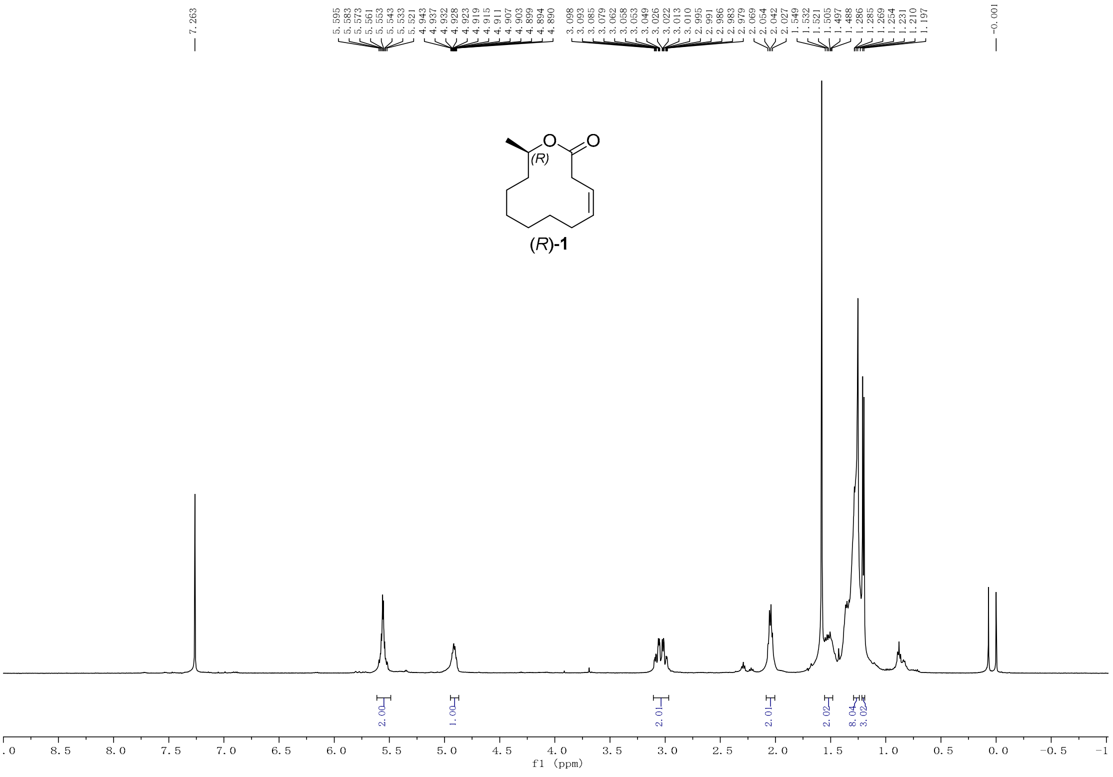

Figure S16. ^{13}C NMR Spectrum of (*R*,*Z*)-12-methyloxacyclododec-4-en-2-one ((*R*)-**1**) (126 MHz, $CDCl_3$).

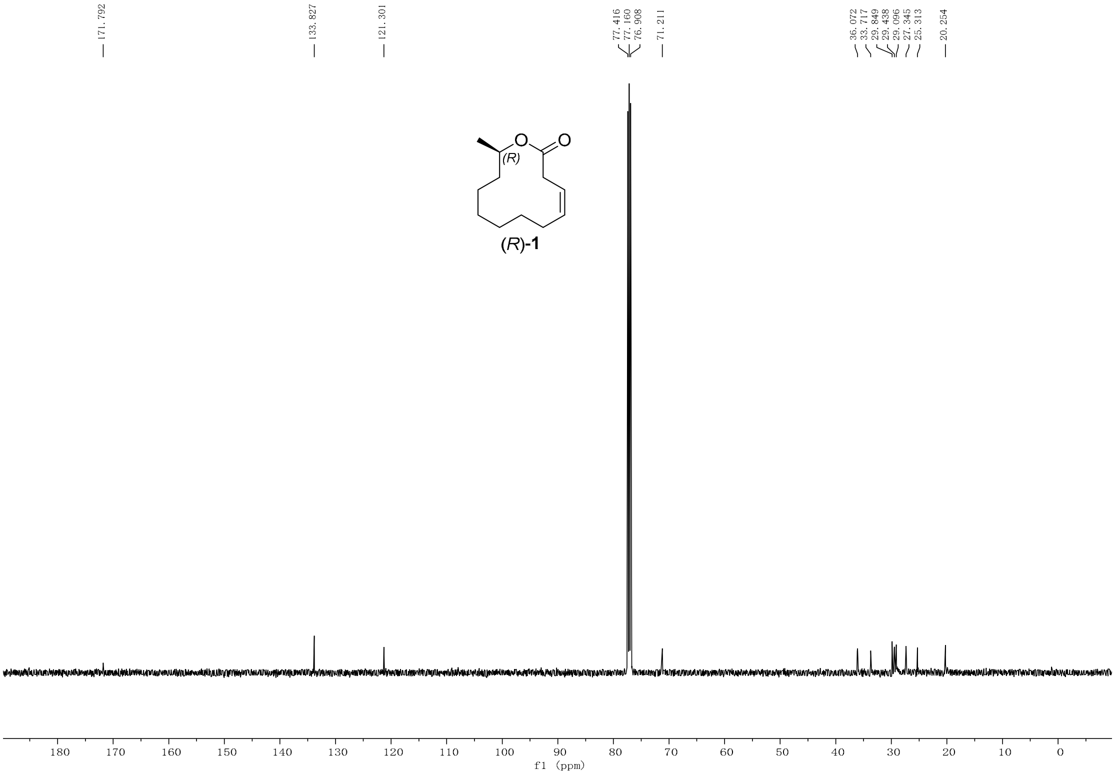

Figure S17. ^{1}H NMR Spectrum of *rac*-dec-3-yn-2-ol (*rac*-**6**) (500 MHz, $CDCl_3$).

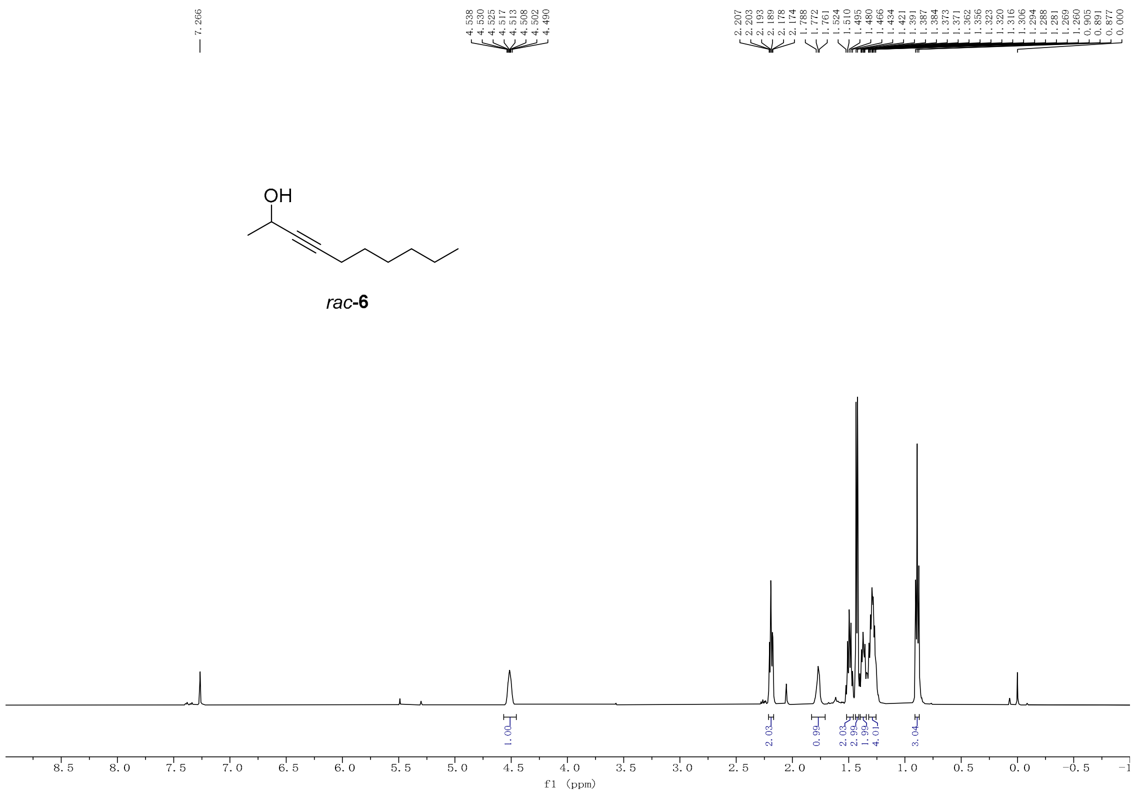

Figure S18. ^{13}C NMR Spectrum of *rac*-dec-3-yn-2-ol (*rac*-**6**) (126 MHz, $CDCl_3$).

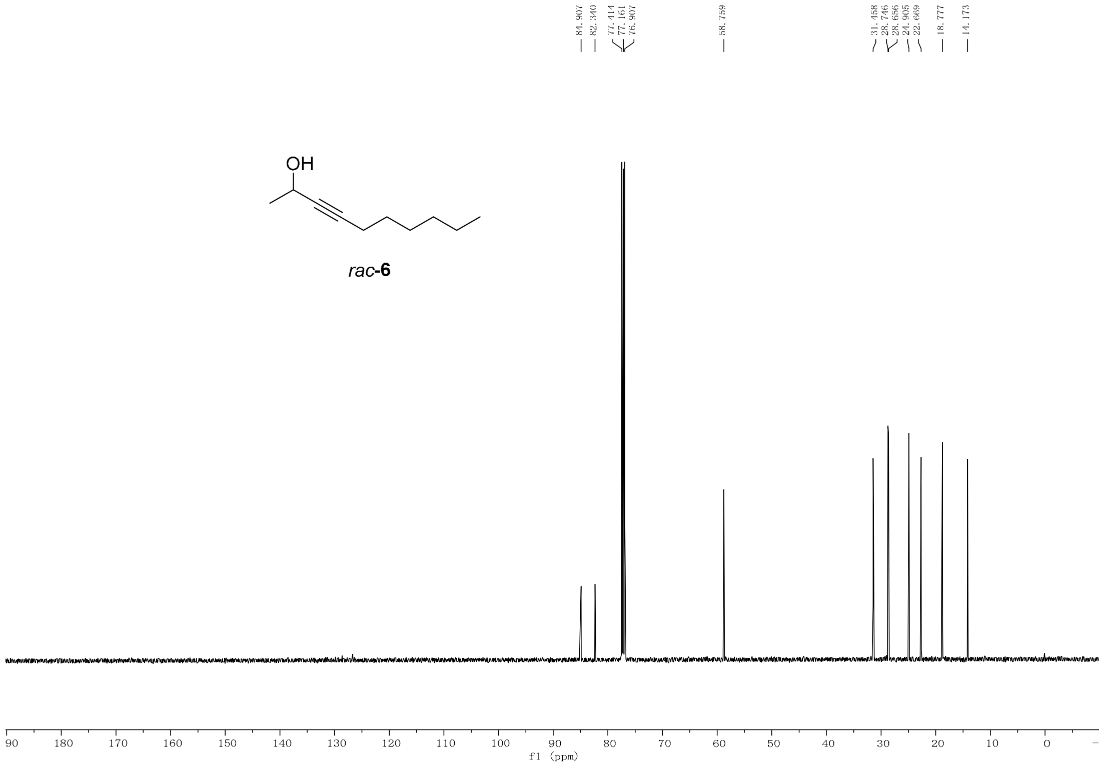

Figure S19. ^{1}H NMR Spectrum of *rac*-dec-3-yn-2-yl 3,5-dinitrobenzoate (*rac*-**14**) (500 MHz, $CDCl_3$).

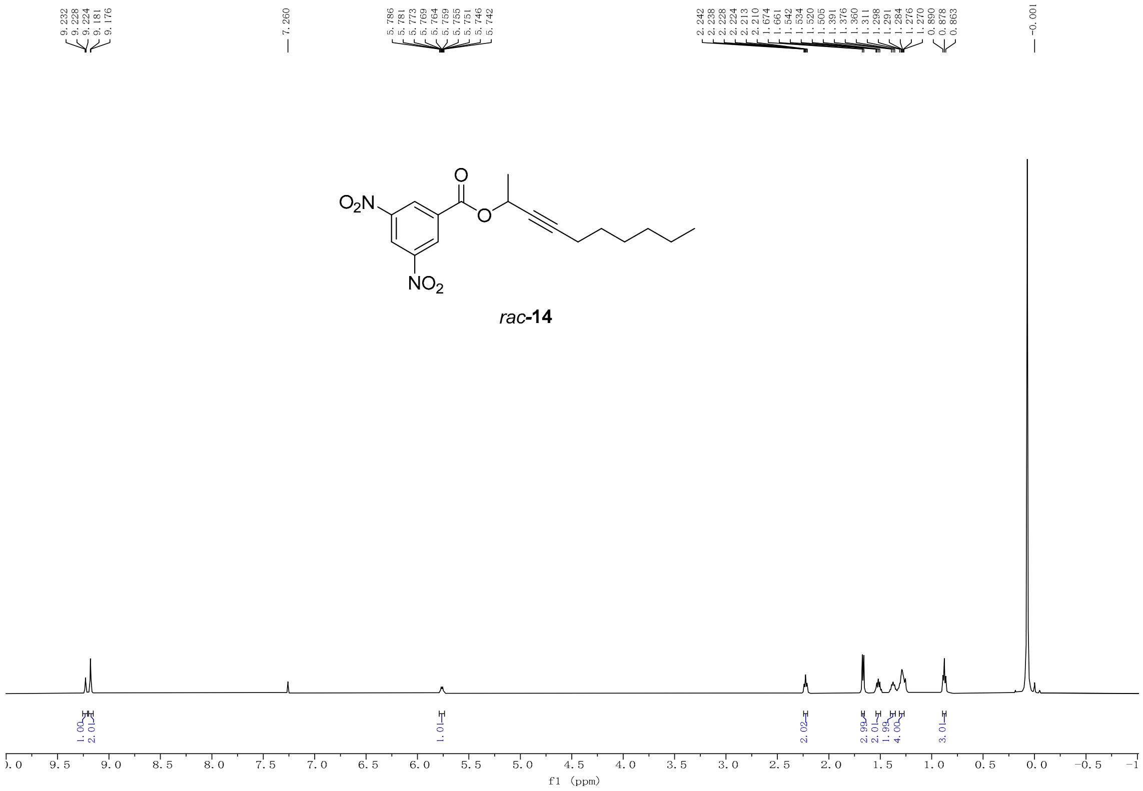

Figure S20. ^{13}C NMR Spectrum of *rac*-dec-3-yn-2-yl 3,5-dinitrobenzoate (*rac*-**14**) (126 MHz, $CDCl_3$).

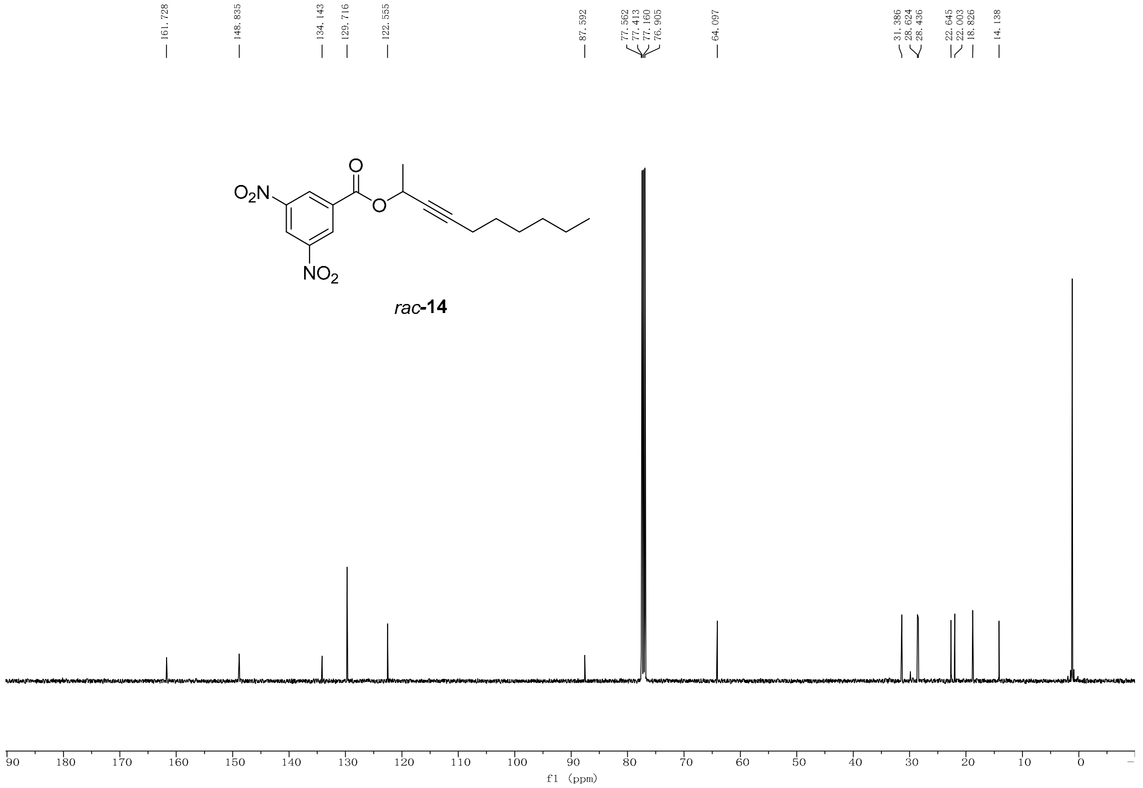

Figure S21. ^{1}H NMR Spectrum of (*S*)-dec-3-yn-2-yl 3,5-dinitrobenzoate ((*S*)-**14**) (500 MHz, $CDCl_3$).

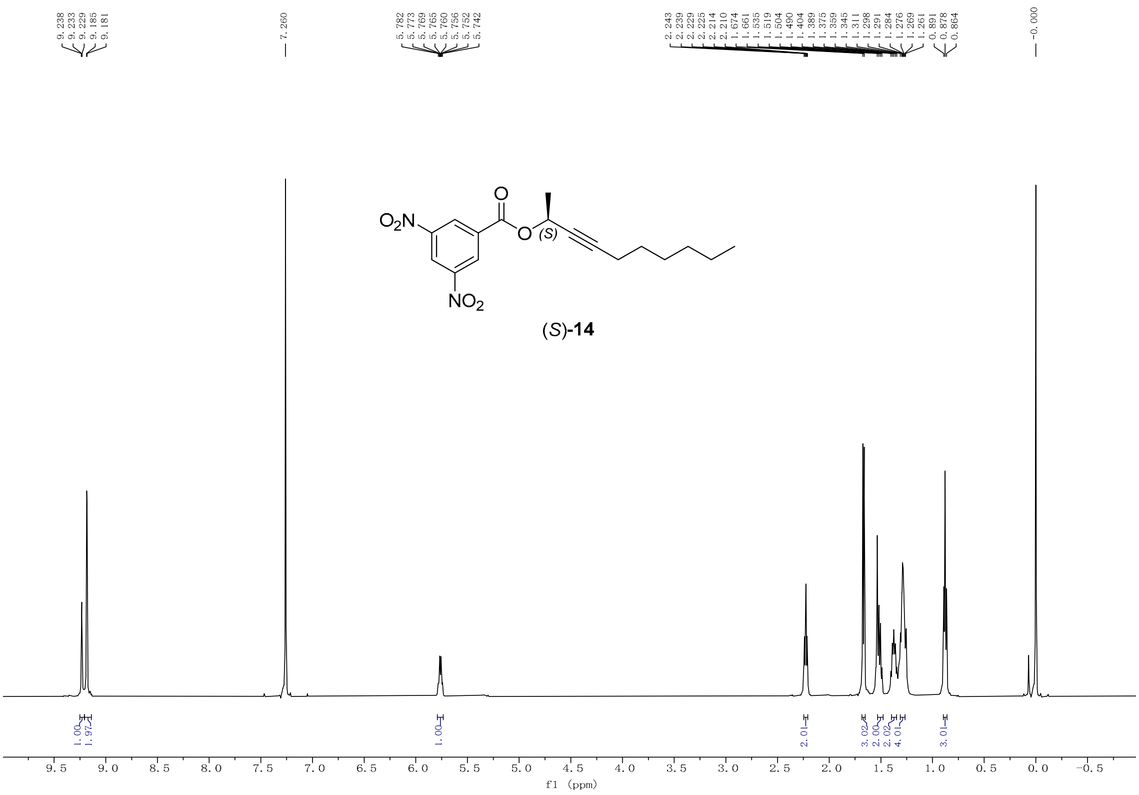

Figure S22. ^{13}C NMR Spectrum of (*S*)-dec-3-yn-2-yl 3,5-dinitrobenzoate ((*S*)-**14**) (126 MHz, $CDCl_3$).

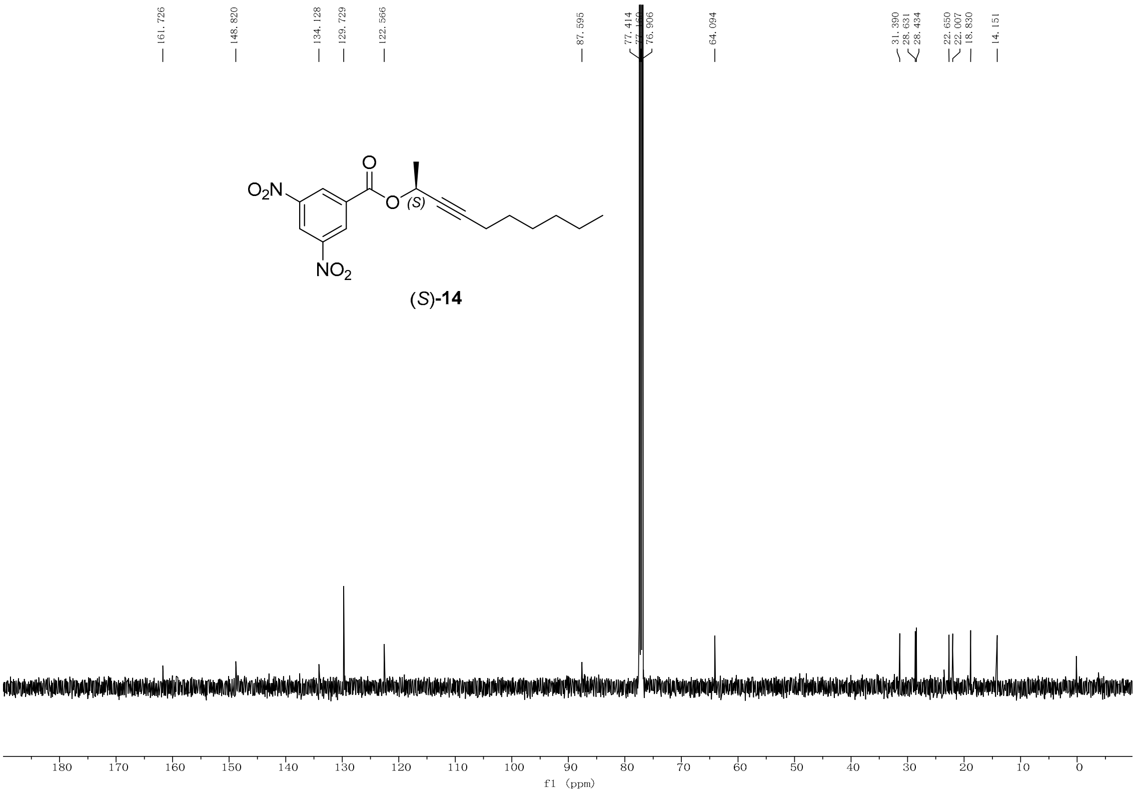

4. HPLC Chromatography and ECD spectra of the Compounds

Figure S23. HPLC Chromatography of *rac*-dec-3-yn-2-yl 3,5-dinitrobenzoate (*rac*-**14**) (Chiralpak AD-H column; *n*-hexane/*i*-propanol = 97:3, 1.0 mL/min, 254 nm).

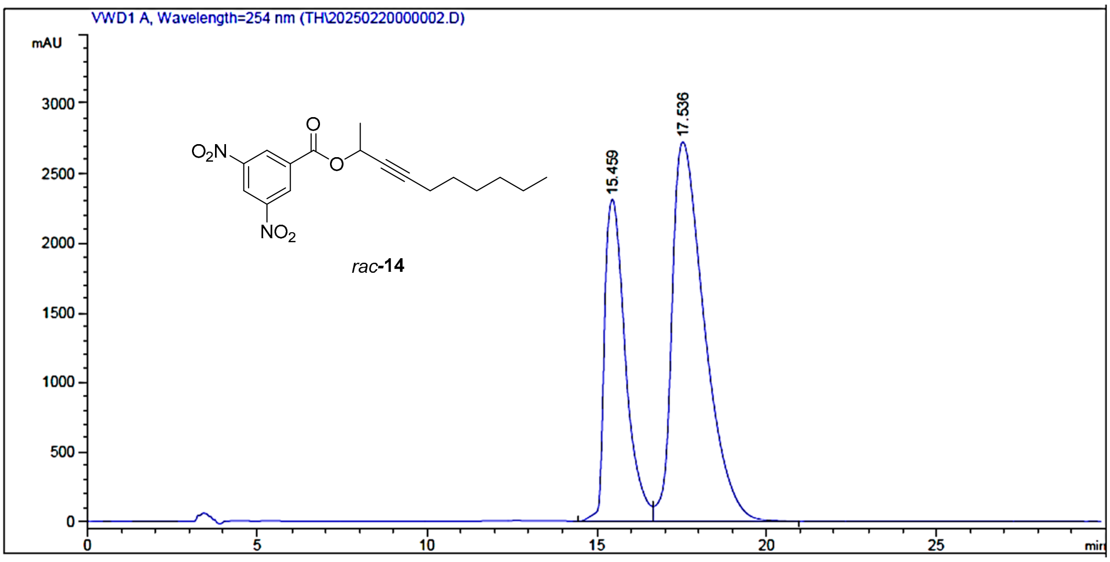

Peak	RetTime	Type	Width	Area	Height	Area
#	[min]		[min]	mAU*s	[mAU]	%
1	15.459	BV	0.6812	9.92571e4	2312.92090	35.3909
2	17.536	VB	0.9969	1.81203e5	2725.64355	64.6091

Figure S24. HPLC Chromatography of (*S*)-dec-3-yn-2-yl 3,5-dinitrobenzoate ((*S*)-**14**) (Chiralpak AD-H column; *n*-hexane/*i*-propanol = 97:3, 1.0 mL/min, 254 nm).

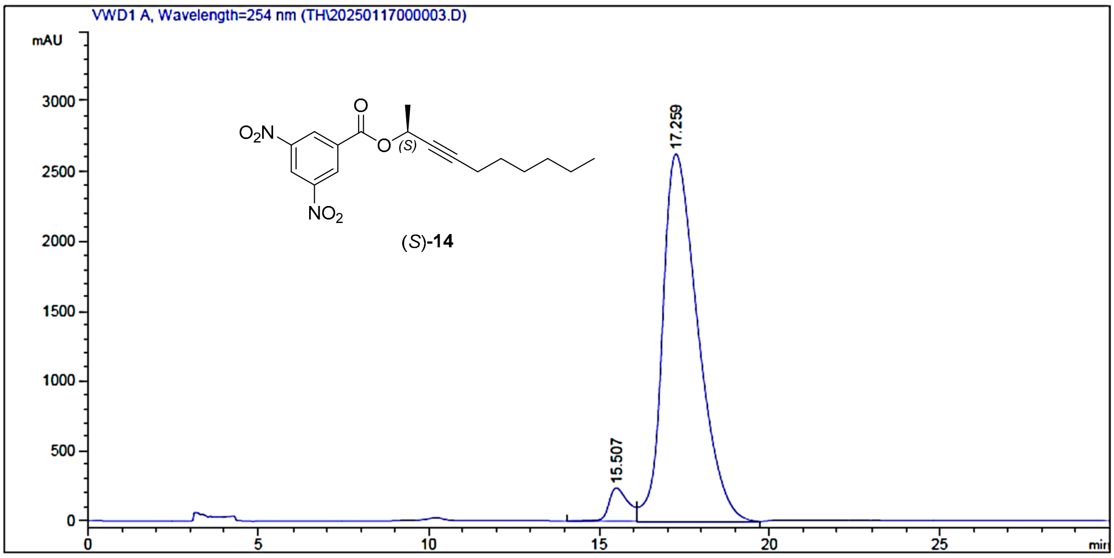

Peak	RetTime	Type	Width	Area	Height	Area
#	[min]		[min]	mAU*s	[mAU]	%
1	15.507	VV	0.5879	9438.72070	237.99547	4.7694
2	17.259	VV	1.0954	1.88464e5	2629.22681	95.2306

Figure S25. Experimental and calculated ECD spectra of (*S*)-**1** and (*R*)-**1**.

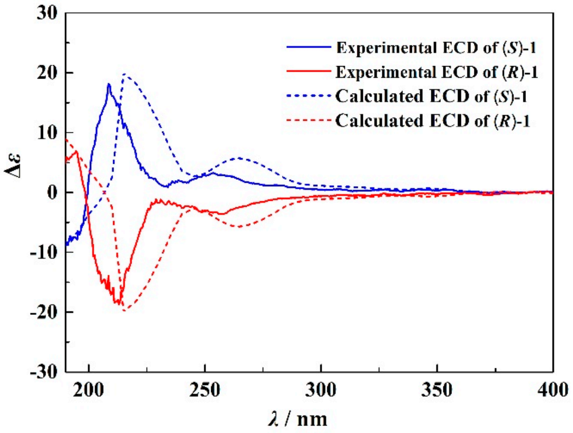

5. References

1. Miura, K.; Wang, D.; Matsumoto, Y.; Hosomi, A. Highly regio- and stereoselective hydrostannylation of alkynols with a new lewis acidic hydrostannane. *Org. Lett.* **2005**, *7*, 503–505.
2. Lin, C.; Zhu, W.; Wu, S.; Bian, Q.; Zhong, J. Asymmetric synthesis and biological activity of contact pheromone of western flower thrips, frankliniella occidentalis. *Int. J. Molecular Sci.* **2024**, *25*, 11699.
3. Harada, S.; Takita, R.; Ohshima, T.; Matsunaga, S.; Shibasaki, M. Ligand accelerated indium(III)-catalyzed asymmetric alkynylation of aldehydes with 2-methyl-3-butyn-2-ol as an ethyne equivalent donor. *Chem. Commun.* **2007**, 948–950. https://doi.org/10.1039/B614958H.
